# Barley heads east: Genetic analyses reveal routes of spread through diverse Eurasian landscapes

**DOI:** 10.1371/journal.pone.0196652

**Published:** 2018-07-18

**Authors:** Diane L. Lister, Huw Jones, Hugo R. Oliveira, Cameron A. Petrie, Xinyi Liu, James Cockram, Catherine J. Kneale, Olga Kovaleva, Martin K. Jones

**Affiliations:** 1 McDonald Institute for Archaeological Research, University of Cambridge, Cambridge, United Kingdom; 2 The John Bingham Laboratory, NIAB, Cambridge, United Kingdom; 3 Manchester Institute of Biotechnology, School of Earth and Environmental Sciences, University of Manchester, Manchester, United Kingdom; 4 Department of Archaeology, University of Cambridge, Cambridge, United Kingdom; 5 Department of Anthropology, Washington University in St. Louis, St. Louis, MO, United States of America; 6 N.I. Vavilov Research Institute of Plant Industry, St. Petersburg, Russia; Saint Mary's University, CANADA

## Abstract

One of the world’s most important crops, barley, was domesticated in the Near East around 11,000 years ago. Barley is a highly resilient crop, able to grown in varied and marginal environments, such as in regions of high altitude and latitude. Archaeobotanical evidence shows that barley had spread throughout Eurasia by 2,000 BC. To further elucidate the routes by which barley cultivation was spread through Eurasia, simple sequence repeat (SSR) analysis was used to determine genetic diversity and population structure in three extant barley taxa: domesticated barley (*Hordeum vulgare* L. subsp. *vulgare*), wild barley (*H*. *vulgare* subsp. *spontaneum*) and a six-rowed brittle rachis form (*H*. *vulgare* subsp. *vulgare* f. *agriocrithon* (Åberg) Bowd.). Analysis of data using the Bayesian clustering algorithm InStruct suggests a model with three ancestral genepools, which captures a major split in the data, with substantial additional resolution provided under a model with eight genepools. Our results indicate that *H*. *vulgare* subsp. *vulgare* f. *agriocrithon* accessions and Tibetan Plateau *H*. *vulgare* subsp. *spontaneum* are closely related to the *H*. *vulgare* subsp. *vulgare* in their vicinity, and are therefore likely to be feral derivatives of *H*. *vulgare* subsp. *vulgare*. Under the eight genepool model, cultivated barley is split into six ancestral genepools, each of which has a distinct distribution through Eurasia, along with distinct morphological features and flowering time phenotypes. The distribution of these genepools and their phenotypic characteristics is discussed together with archaeological evidence for the spread of barley eastwards across Eurasia.

## Introduction

This paper presents a study of the prehistoric spread of domesticated barley (*Hordeum vulgare* L. subsp. *vulgare*) across Eurasia, using SSR marker analysis. The aims of this study are to: (1) discern routes of spread through patterns in population structure, and to compare these routes with relevant archaeobotanical evidence; (2) to examine the geographical partitioning of this population structure in relation to morphological traits and genotypes of flowering time genes, in order to understand the roles of environmental adaptation and human choice in the spread and establishment of barley cultivation; and (3) to examine the relationship between the domesticated and wild sub-species of *H*. *vulgare*.

Barley is one of the world’s most important and resilient crops, able to grow in marginal environments where other crops are unable to grow, which has important implications for food security [1]. Domesticated barley comprises both 2-row and 6-rowed spike, and hulled and naked caryopsis forms, each which continue to be cultivated on significant scales. The material analysed in this study consists of well-provenanced cultivated barley accessions (*H*. *vulgare* subsp. *vulgare*; hereafter referred to as *vulgare*), which are described in germplasm collections as ‘landraces’ (varieties circulating among kin and village groups, rather than acquired commercially from markets or crop breeders). These landraces are from across Asia, along with two brittle-rachised sub-species that various authors have claimed association with the ancestry of domesticated barley: 2-rowed *H*. *vulgare* subsp. *spontaneum* (hereafter referred to as *spontaneum*) from the Near East, Central Asia and the Tibetan Plateau; and 6-rowed *H*. *vulgare* subsp. *vulgare* f. *agriocrithon* (Åberg) Bowd. (hereafter referred to as *agriocrithon*) from Central Asia, China and the Tibetan Plateau [2, 3]

The emergence and spread of domesticated plants and animals in a number of regions of the world around 11,000 years ago was one of the most important transitions in the development of human societies. Barley was one of the earliest domesticated crops, emerging in the Near East around 11,000 years ago. By 2,000 BC, barley cultivation had spread to Europe, North Africa, South Asia and East Asia. Today barley is the world’s fourth most important cereal crop, after wheat, rice and maize. The Eurasian continent contains a diverse array of landscapes, from the world’s highest mountains and driest deserts, and spans from the Arctic to the Tropics, which presents an ideal opportunity to look at aspects of the spread of barley cultivation in the context of ecological adaptation.

### The archaeobotanical evidence

Archaeological evidence from southwest Asia shows that two-rowed brittle-rachis wild barley was being used for at least 10,000 years before the fixation of the tough rachis trait [4, 5]; this trait is often taken as the marker of ‘domestication’ (e.g. [6]). Macrofossils of two-rowed barley with a tough rachis were first identified at Tell Abu Hureyra, Syria, in the early 9^th^ millennium BC [7]. At Ali Kosh, in Iran, six-rowed barley with a tough rachis was identified in the 8th millennium BC, with naked caryopsis forms from the 7^th^ millennium BC [8].

Subsequently, domesticated barley moved out of the Near East into Europe, North Africa, and Central, South and East Asia. In Europe, domesticated barley first appears at sites in the Aegean from the ninth to the 7^th^ millennium BC [9, 10]. From there it spread along a northern trajectory through Central Europe, following the Danube and Rhine river valleys through central Europe, into the North European Plain, with further dispersals into the British Isles and Scandinavia; e.g. barley cultivation reaches the Arctic Circle by the late Bronze Age (1,000 to 800 cal. BC) in the site of Kveøy in Norway [11]. Barley cultivation also spread by a southerly route along the Mediterranean coast through Italy to Iberia; e.g. barley is present in the NE Iberian Peninsula by 5,400 cal. BC in sites such as Balma Margineda [12]. In North Africa, the earliest barley, with naked forms, was in Morocco by 5,500 cal. BC at sites such as Ifri Oudadane. The lack of evidence for farming in other regions of North Africa suggests that the Southwest Asia crops could have arrived in the area through a maritime route, most likely from Italy or the Iberian Peninsula [13].

Turning to the eastward spread of barley cultivation through Eurasia, six-rowed barley was the predominant crop at Mehrgarh, a key site seen to be precursor to the Indus civilization, situated at the boundary of the Iranian Plateau and the Indus flood plains, from the 7^th^ millennium BC [14]. Both hulled and naked types are found at archaeological sites dating to the 3^rd^ and 2^nd^ millennia cal. BC across northern and central India, and in southern India by the late 1^st^ millennium cal. BC [15]. From evidence at sites such as Ojakly in Turkmenistan, barley had spread further east into Central Asia by the mid 2^nd^ millennium cal. BC [16]. Until recently, little archaeobotanical data was available in the large region between the Caucasus and the Hexi Corridor in western China; however, with the use of flotation to recover plant remains, authors such as Spengler et al. [17, 18], Frachetti et al. [19, 20] and Stevens et al. [21] have published data showing the presence of barley at sites in this region. Spengler [22] has reported that there was a gradual shift towards naked barleys within Central Asia over a period of at least two thousand years. By the 3^rd^ millennium cal. BC, barley cultivation was widespread in many parts of China [15], and had reached Korea [23] and middle Jomon Japan [24].

Zhao [25] has proposed three different routes for the eastwards spread of wheat (and by inference barley, which is often found alongside wheat in archaeological sites) from the Near East into China around 2,000 BC: (1) the Eurasian Steppe Route, following the vast steppe lands of Central Asia and Mongolia, (2) a Sea Route, whereby the crops were spread into south eastern China by seafarers from the Indus civilization of South Asia, and (3) an inland route via the Hexi Corridor, which in historic periods would become an important route between Central Asia and north China. Frachetti [26] in considering the transmission of crops across Eurasia during the Bronze and Iron Ages, has drawn attention to an ‘Inner Asian Mountain Corridor’ (IAMC), the corridor through the Pamir, Tian Shan and Altai ranges. This corridor reaches the Hexi Corridor further east, which later formed part of the northern Silk Road in China. From their recently published database of archaeobotanical remains and radiocarbon dates, Stevens et al. [21] discuss evidence for the broadly synchronous spread of wheat and barley into China through routes both to the northeast and southeast of the Tibetan Plateau, during the 3^rd^ millennium BC. Liu et al. [15, 27] shows that the earliest direct radiocarbon dates for barley are along the southern side of the Tibetan Plateau are around a thousand years older than those on the northern side, and that the spread of barley is distinct from that of wheat. The oldest dated barley remains in China was found in Qinghai, on the northeastern Tibetan Plateau, dating to the early 2^nd^ millennium cal. BC, at altitudes of at least 3,600 metres above sea level (masl) [28].

### The genetic evidence

The key phenotypic trait that distinguishes the spike of domesticated barley from its wild progenitor is a tough, or non-shattering, rachis. The non-shattering spike ensures efficient harvesting, with minimal loss of grain. Genetic studies have shown that mutations in two adjacent genetic loci are responsible for this phenotype, *Btr1* and *Btr*2, and that the non-brittle phenotype is conferred by recessive alleles at either gene [29]. An additional causal mutation in the *Btr1* gene has recently been reported in a small number of landrace accessions [30]. DNA sequence analysis of wild and domesticated barley accessions showed that the two tough rachis mutants originally identified in barley, are associated with genetically distinct groups of *vulgare*, with distinct eastern and western distributions in the Near East; the authors suggest that this is evidence for at least two domestications of barley [31]. Saisho and Purugganan [32], using sequence data and the distribution of 2/6-rowed and hulled/naked phenotypes, show similar results. The barley domesticated in the western Fertile Crescent was the progenitor of North African and European barleys, and the barley domesticated east of the Fertile Crescent, most probably in the Zagros Mountains of Iran, was the progenitor of East and South Asian barleys. Other authors have proposed that barley was also domesticated in the Western Mediterranean [33] and on the Tibetan Plateau, e.g. [34, 35].

A recent study by Poets et al. [36], using whole genome data, suggests that introgression between wild and domesticated barley has had an important role to play in the current genetic make-up of domesticated barley. Landraces show evidence of introgression from multiple geographically dispersed wild populations. Western wild barley populations have genetically contributed most directly to African and European landraces, while eastern wild barleys have contributed more to Asian landraces. The authors also show that these introgression events are ancient rather than recent, and that they have also played a primary role in the environmental adaptation of cultivated barley, as introgression from proximal wild barley populations contributed to locally adaptive variation.

### Expanding the analysis from Europe to Eurasia

Through the genetic analysis of extant crop landraces we have previously explored the prehistoric spread of barley cultivation into Europe and North Africa, with analysis of SSR markers and DNA sequence data. The results of these studies have demonstrated that there were multiple introductions of cultivated barley into Europe, mapping onto attested routes of Neolithic agricultural spread, e.g. [37–40].

Here we complement this knowledge of the westward prehistoric spread of *vulgare* with evidence of its eastward spread across Eurasia, and its relationship with the brittle-rachis forms found within the Near East and Asia, *spontaneum* and *agriocrithon*. This is achieved through SSR marker analysis of 516 accessions of three barley sub-taxa distributed across the Near East, Central, South and East Asia, and the Tibetan Plateau.

In the context of this analysis, we also consider the role that environmental adaptation played in that spread. The southwest Asian crops were adapted to the hot and dry Mediterranean climate of this region. The spread beyond southwest Asia brought *vulgare* into very different environments, with markedly different day-lengths and altitudes, for example. Necessary adaptations included adjustment of flowering times (e.g. [41]). In order to complete their life cycle it is essential that the flowering of plants coincide with favourable seasonal conditions, avoiding damage to sensitive floral tissues through extremes of temperature or drought [42]. In this study we analysed two sets of flowering time genes that proved informative in the European study: the photoperiod response gene *PHOTOPERIOD 1* (*PPD-H1*), which is involved in flowering time being triggered by long days [42–44], and two vernalization genes, *VERNALIZATION 1* (*VRN-H1*, [45]) and *VERNALIZATION 2* (*VRN-H2* [46]), which control the initiation of flowering after a period of chilling in the vegetative state. Crops requiring a period of chilling are considered to have a winter growth habit, and those that don’t a spring growth habit.

Genetic and genomic studies of cultivated crops and their wild progenitors can yield useful data about the spread and establishment of agriculture. In this study of well-provenanced barley landraces, and related sub-taxa, we can discern a number of routes of spread of barley cultivation, through diverse environments that reflect known movements of people and crops. These data can complement and provide additional resolution to the spread of barley from archaeobotanical data, particularly the recent paper published by Liu et al. [15], which has provided a detailed radiocarbon dating framework using directly dated barley grains, from which to interpret the genetic data presented in this paper.

## Materials and methods

### Plant material and DNA extraction

Details of the 351 *vulgare*, 142 *spontaneum* and 23 *agriocrithon* accessions are listed in S1 Table, including the donor institutes from which the materials were obtained. Included are 25 *vulgare* accessions that were field collected in China by our project team along the edge of the Tibetan Plateau in Gansu and Qinghai provinces, from altitudes ranging between 2755 to 3164 masl. Where possible, germplasm accessions were selected that have precise geographical collection site data. *Spontaneum* accessions were selected from throughout its range in the Near East, Central Asia and on the Tibetan Plateau. *Agriocrithon* accessions were from Central Asia and China, including the Tibetan Plateau. For *vulgare* accessions, available passport data on row number and caryopsis structure (hulled or naked grains) was recorded, and additionally determined using visual inspection (S1 Table).

DNA was extracted from leaf material harvested from a single individual per accession using a modified Tanskley method [47] or using a Qiagen DNeasy Plant Mini Kit (Qiagen), according to the manufacturer’s instructions. The DNA concentration was determined using a Qubit 2.0 fluorometer (Invitrogen) and adjusted to 10 ng/μl.

### Genotyping

#### SSR genotyping

Nineteen simple sequence repeats (SSR) markers were genotyped in all *vulgare*, *spontaneum* and *agriocrithon* accessions, using the primer pairs listed in S2 Table. These SSRs were selected to allow data integration with previous investigations of population structure in European *vulgare* and Near Eastern *spontaneum* accessions [40]. Further details are in S1 Text.

#### Molecular determination of the seasonal growth habit phenotype

We predicted seasonal growth habit (SGH) for a subset of accessions (232) using PCR-based assays for mutations in the vernalization genes *VRN-H1* and *VRN-H2* [46, 48]. Details are given in S1 Text.

#### *PPD-H1* genotyping

The identity of the putative causative non-synonymous single nucleotide polymorphism (SNP) T+2036/C SNP (Thr → Ala) in *PPD-H1* exon 6, was assayed (SNP position relative to the start codon of GenBank accession AY943294; [41]) using Sanger sequencing and KASP genotyping (LGC Genomics, Hoddeson, U.K.). Details are given in S1 Text.

### Data analysis

#### Population structure and geo-plotting

A joint dataset of all *vulgare*, *spontaneum* and *agriocrithon* accessions was analysed using 19 SSRs. Barley has a high self-pollinating rate of over 98% [49]. Thus, we used InStruct software [50], which implements a similar clustering algorithm to the software STRUCTURE [51], but does not assume Hardy–Weinberg equilibrium. As InStruct does not accept haploid input data, a false-diploid dataset was created by duplication of each allele. The number of homogeneous genepools (*K*) between one (*K* = 1) and ten (*K* = 10) was modeled with a burn-in of 500,000 and 1,000,000 Markov Chain Monte Carlo (MCMC) runs, using the admixture model, with 20 replications for each value of *K*. The best-fit model was determined by plotting the natural log probability of the data against *K* [52]. Correlations of Q matrix output among replicate runs were determined using CorrSieve 1.6–8 [53].

Accessions were geographically mapped using the longitudes and latitudes reported in by germplasm collections, where this information was available, or estimated coordinates from other provided locality details using Google Earth. Maps were drawn using ArcMap v. 10.2. Topographic base maps were from NASA Blue Marble: Next Generation satellite imagery, originally produced by Reto Stöckli and obtained from NASA’s Earth Observatory (NASA Goddard Space Flight Center); http://earthobservatory.nasa.gov/Features/BlueMarble/.

Population structure in the three barley taxa was further investigated by principal component analysis (PCA), which was calculated and plotted with the R environment [54], for statistical computing using the packages *FactoMiner* [55] and *ggplot2* [56], respectively.

#### Genetic diversity

Nei’s pairwise genetic distance (*D*) [57], between groups of accessions and individual accessions, was calculated using PowerMarker [58], based on the allele frequencies for 19 SSRs. In these analyses, individual accessions were assigned to the genepool with the highest proportional membership (> 50%). Pairwise distance matrices between groups were also calculated using PowerMarker, using *Nei72*. This distance matrix was used as input data for the *gplots* R package [59], and a heat map produced using the function *heatmap*.*2* [60]. The diversity statistics produced included expected and observed heterozygosity (*H*_*E*_ and *H*_*O*_), number of alleles (*N*_*A*_ and inbreeding coefficient (fixation index, *F*). Diversity was compared between different subspecies and InStruct genepools at *K* = 8.

#### Phylogenetic analysis

Relationships among the accessions were described by calculating their shared allele distances and subsequently using these to draw a Neighbour-Joining (N-J) tree using the *prabclus* package (Functions for clustering of presence-absence, abundance and multilocus genetic data) in R [60].

## Results

### Genotyping and genetic diversity in the three barley taxa

Raw genotyping data files generated using InStruct are in S1 file.zip. Detailed information about SSR scoring is detailed in S1 Text. Genotyping scoring data is recorded in S3 Table. The number of alleles detected in this dataset was 261. Q matrices from InStruct analysis are in S4 Table; marker diversity statistics are in S5 Table. The numbers of alleles per locus varied from 5 (M29) to 38 (M9), with a mean of 13.7. Only SSR marker M13 showed heterozygosity. Marker diversity was lowest in *agriocrithon*, with all markers having 6 or fewer alleles, except for M9, which had 8. For *spontaneum*, the number of alleles varied from 4 (M20 and M29) to 27 (M9). A number of markers performed poorly in some accessions, with missing data frequencies varying from 2 to 33% (mean 9%).

#### Genetic diversity in the three barley taxa

Total genetic diversity (*H*_*E*_) was analysed for each taxa (S6 Table). *Spontaneum* accessions showed the highest level of diversity (*H*_*E*_ = 0.724), whereas *agriocrithon* and *vulgare* showed similar levels (*H*_*E*_ = 0.538 and 0.594, respectively). Observed heterozygosity (*H*_*O*_) was consistently lower in each group (0.260 to 0.031), and *F* values were consistently high (0.955 to 0.956).

**Fig 1:**
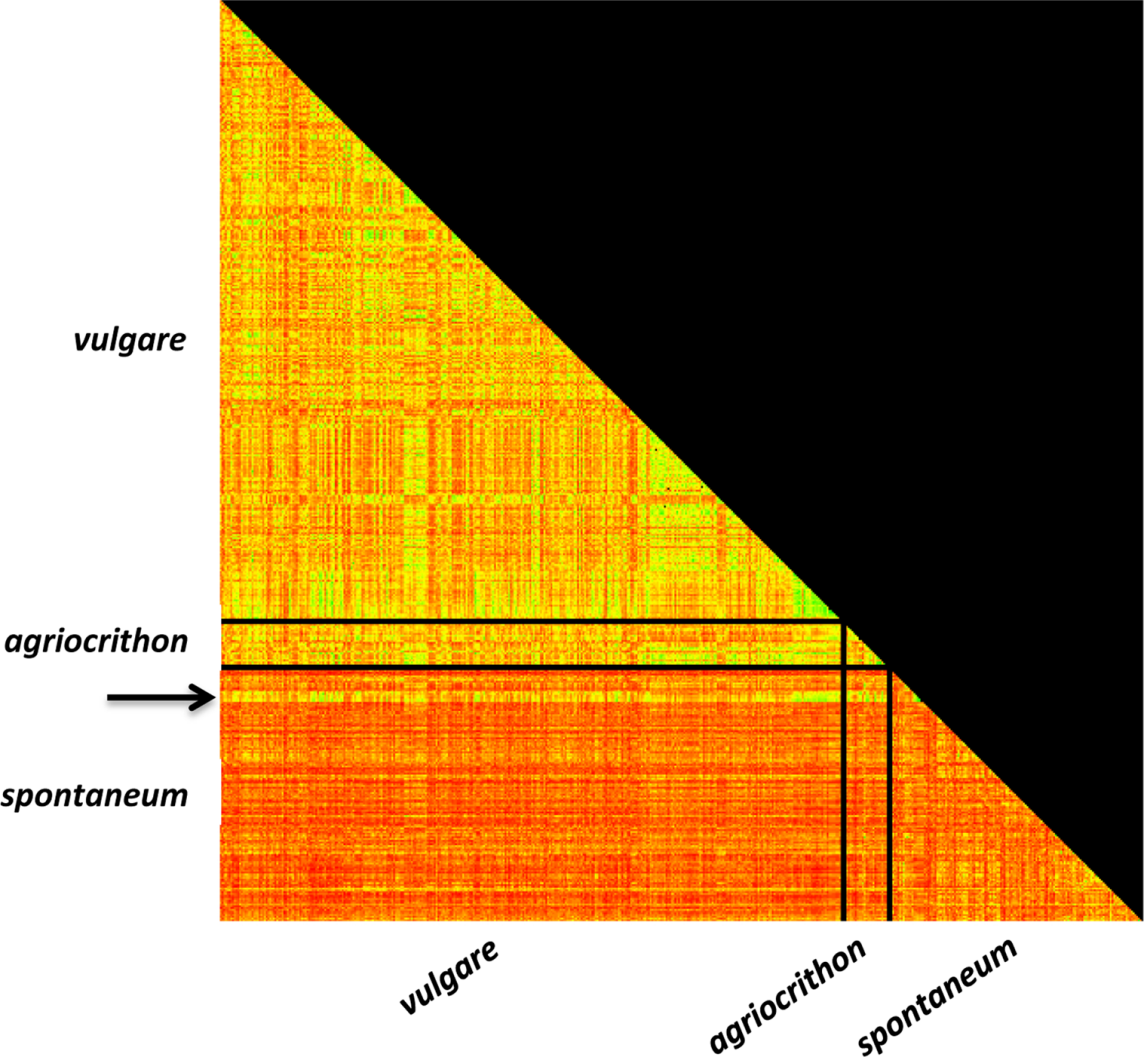
Heat map of pairwise genetic distances between three barley taxa. Analysis included 351 *vulgare*, 142 *spontaneum* and 23 *agriocrithon* accessions, and was based on the allele frequencies of 19 SSR markers. Genetic distances were calculated with GenAlEx based on Nei's pairwise genetic distance (*D*) [57]. The paler colour (yellow) represents closer genetic distances, whereas darker colours (red) represent more distant relationships. The arrow indicates the Tibetan *spontaneum* accessions, which are genetically closer to all the cultivated barley accessions than to other *spontaneum* accessions.

In pairwise comparisons of genetic diversity were undertaken (S7 Table), the comparison of *vulgare* with *spontaneum* and *agriocrithon* resulted in a low fixation index (*F*_*ST*_ = 0.031), indicating greater similarity between *vulgare* and *agriocrithon* accessions, than between *spontaneum* and *agriocrithon* (*F*_*ST*_ = 0.087), and *spontaneum* and *vulgare* (*F*_*ST*_ = 0.075). Nei’s pairwise genetic distance (*D*) plotted as a heat map also supported a more distant relationship between *spontaneum* and the other two taxa and a high degree of relatedness between *agriocrithon* and *vulgare*, and *vulgare* and the six Tibetan *spontaneum* accessions (Fig 1).

### Investigation of population structure in *vulgare*, *spontaneum* and *agriocrithon*

**Fig 2:**
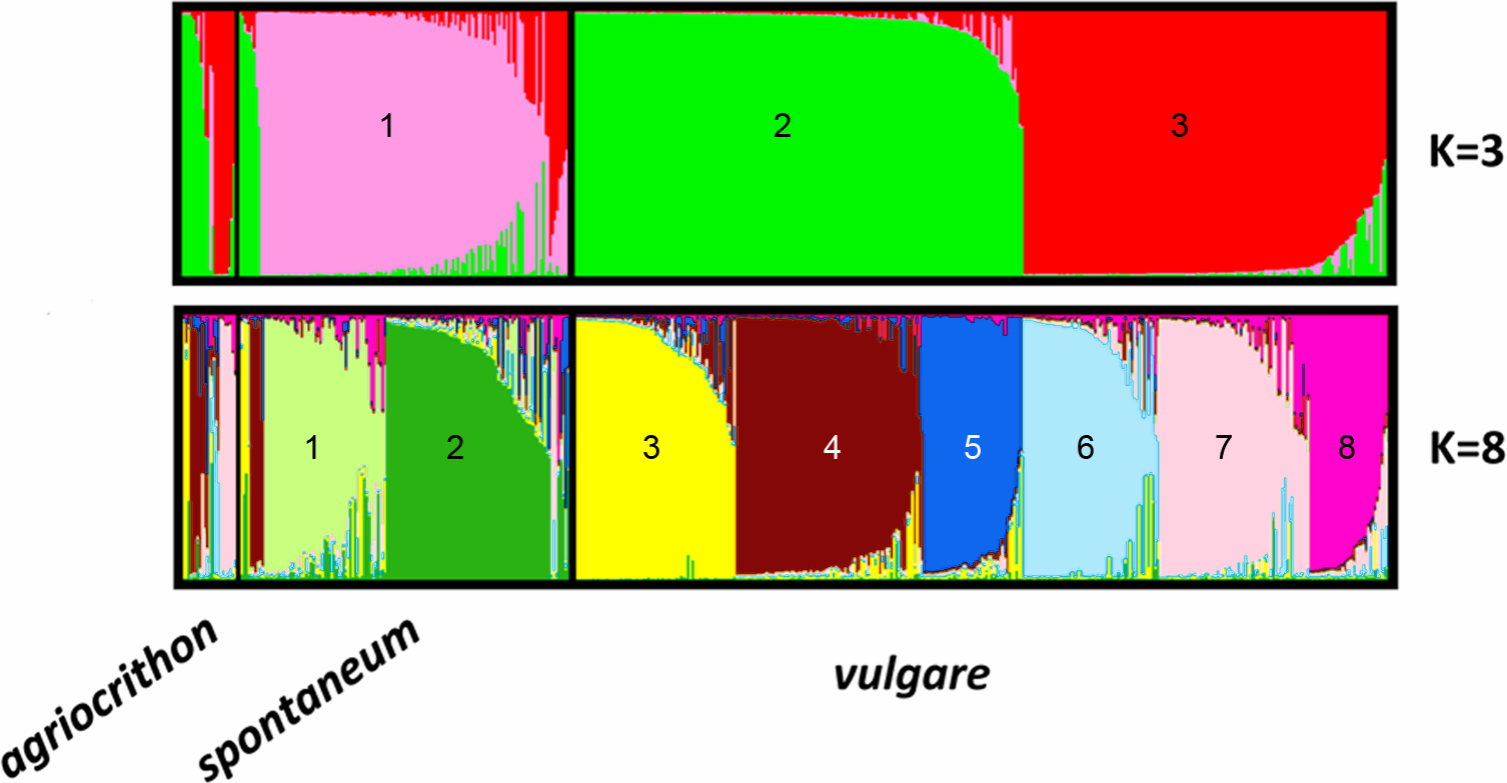
Clustering of accessions of three barley taxa using InStruct analysis of SSR markers. Based on allele frequencies for 19 SSR markers in 351 *vulgare*, 142 *spontaneum* and 23 *agriocrithon* accessions. Plots are of the most likely number of genepools, (*K* = 3 and *K* = 8) using probability measures and *ΔK* analysis (see S1 Fig). Each accession is represented by a vertical line with a proportion of its alleles derived from each modeled genepool, which are each represented by different colours. Accessions are ordered by taxon and then genepool.

InStruct analysis of all datasets revealed population structure among accessions with a degree of admixture between clusters. Individuals were assigned to the genepool with which they had > 50% membership. Analyses of *ΔK*, *LnP(D)* and *Q*-matrix correlations **(**S8 Table and S1 Fig) indicated that *ΔK* values for *K* = 3 and *K* = 8 were significant. The two models relate to one another hierarchically (Fig 2): genepool *K*3_1 (pink; almost exclusively *spontaneum*) at *K* = 3 is broadly subdivided into two at *K* = 8, *K*8_1 and *K*8_2. Genepools *K*3_2 and *K*3_3 (green and red), which are predominantly *vulgare*, along with most of the *agriocrithon* accessions, broadly subdivide into six at *K* = 8 (*K*8_3 to *K*8_8). The plots of *LnP(D)* and *ΔK* against *K* suggest that a model with 3 genepools captures a major split in the data, with substantial additional resolution provided under a model with *K* = 8. Further details of the hierarchical structure of the two models are described in S1 Text.

**Fig 3:**
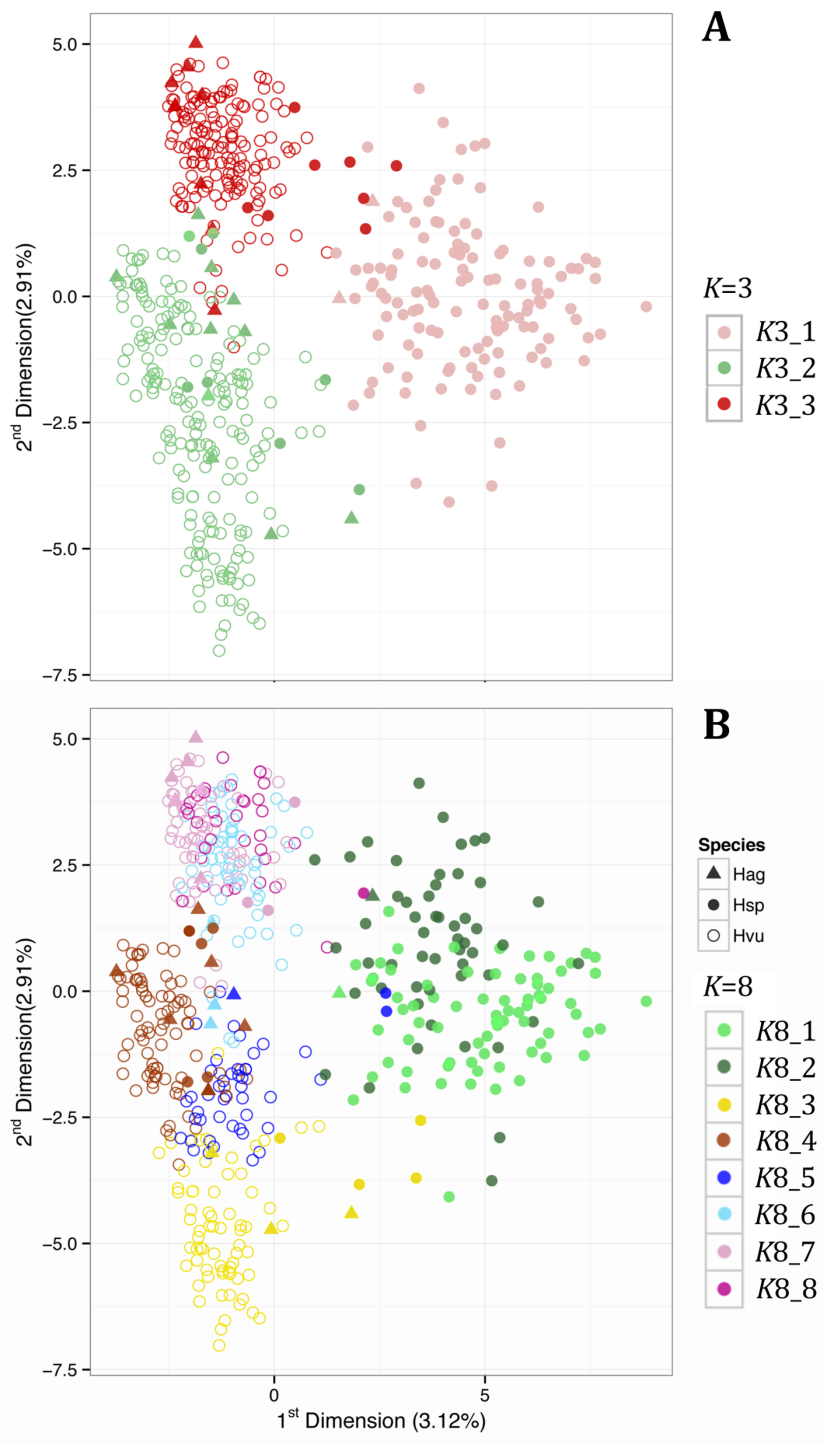
Principle component analyses (PCAs) of individual accessions characterised by 19 SSR markers. The results are based on allele frequencies for 19 SSR markers in 351 *vulgare*, 142 *spontaneum* and 23 *agriocrithon* accessions. Plots are of the first two components at (A) *K* = 3 and (B) *K* = 8 genepools. Each dot represents an accession (open circles, *vulgare*; closed circles, *spontaneum*; triangles, *agriocrithon*), coloured according to the genepool with the highest proportional membership ascribed in the *K* = 8 InStruct model.

PCA found the first two principal components explained 3.1% and 2.9% of the variation, respectively (Fig 3). Unlike the case in elite barley cultivars (e.g. [61]), the major agronomic traits ear-row number (2 or 6 row), SGH (spring or winter), long-day photoperiod response (responsive or non-responsive) and caryopsis type (hulled or naked) were not partitioned between the major InStruct clusters at *K* = 3 (S1 Table); further details of accession phenotypes and their genepool designation is listed in Table 1 and in S1 Text. At *K* = 3, the first principle component essentially separated *spontaneum*, genepool *K*3_1, from *vulgare*/*agriocrithon*, *K*3_2 and *K*3_3 (Fig 3A). The second principle component resolved two main clusters, each consisting of mixtures of *vulgare* and *agriocrithon* accessions, with the upper and lower clusters corresponding to InStruct genepools *K*3_3 and *K*3_2, respectively. Overlaying results for *K* = 8 found the two *spontaneum* sub-populations (*K*8_1 and *K*8_2) to occupy mostly separate PCA spaces. Furthermore, the uppermost *vulgare*/*agriocrithon* PCA cluster comprised three overlapping genepools (*K*8_6, *K*8_7 and *K*8_8), while the lower PCA cluster constituted three almost discrete genepools (*K*8_3, *K*8_4, and *K*8_5) (Fig 3B).

**Table 1:** Phenotypic data for the six *vulgare* genepools at *K* = 8.

Genepool	No. in genepool	Caryopsis (H:N)	Row type (6:2)	Predicted SGH (S:W)	*PPD-H1* (C:T)
***K8_3***	69	55:12 82:18%	63:1 98:2%	17:51 25:75%	65:0 100:0%
***K8_4***	81	5:75 6:94%	72:2 97:3%	10:51 16:84%	67:8 89:11%
***K8_5***	43	41:2 95:5%	43:0 100:0%	32:7 82:18%	42:0 100:0%
***K8_6***	59	56:3 95:5%	17:33 34:66%	35:24 59:41%	12:46 21:79%
***K8_7***	65	62:1 98:2%	45:12 79:21%	52:7 88:12%	15:42 26:74%
***K8_8***	34	33:1 97:3%	29:2 94:6%	23:8 74:26%	30:3 91:9%

Genepool designations are based on allele frequencies for 19 SSR markers in 351 *vulgare*, 142 *spontaneum* and 23 *agriocrithon* accessions, as determined by InStruct analysis. The second column indicates the numbers of *vulgare* accessions in each genepool. Data for caryopsis type (H = hulled, N = naked) and row type (6 = six-rowed, 2 = two-rowed) are derived from passport data and visual inspection. Predicted SGH (S = spring, W = winter) was determined using PCR based assays of the vernalization genes *VRN-H1* and *VRN-H2* (see S1 Text). The causative *PPD-H1* SNP according to [41] was determined using KASP genotyping and Sanger sequencing (C = wild-type, flowering promoted in response to long days, T = non-responsive to long days; see S1 Text). Numbers of each type were determined, and expressed as percentages. Note–phenotypes data for some accessions were not available.

**Fig 4:**
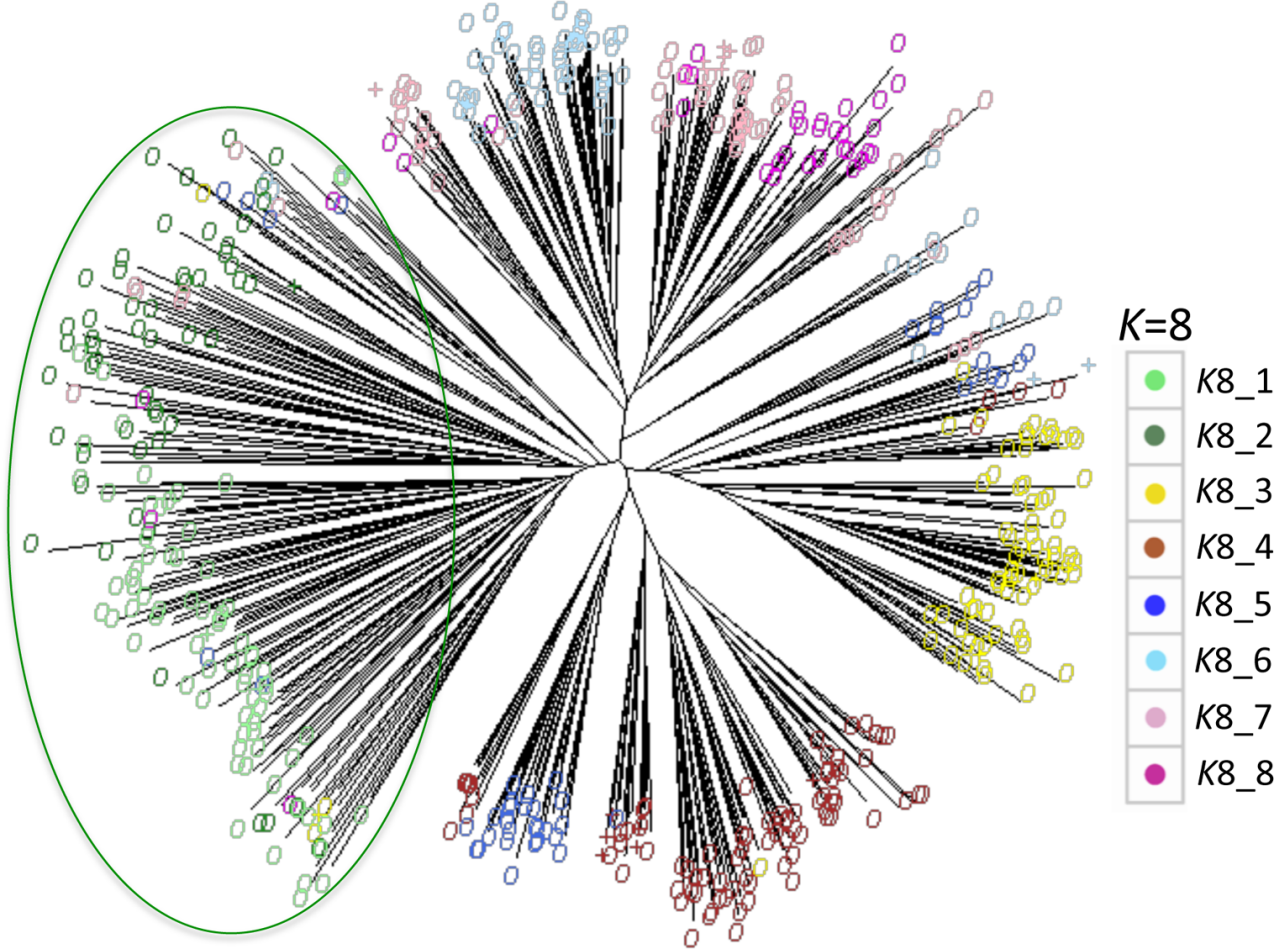
Neighbour joining (N-J) tree constructed from SSR genotypes of three barley taxa. Based on allele frequencies for 19 SSR markers in 351 *vulgare*, 142 *spontaneum* and 23 *agriocrithon* accessions, which were analysed using Nei’s genetic distance, and drawn using the *prabclus* package [60]. Accessions are coloured according to the genepool with the highest proportional membership ascribed in the *K* = 8 InStruct model (> 50%). *Agriocrithon* accessions are indicated as ‘+’. The green ellipse surrounds the *spontaneum* accessions.

The relationship between genepools at *K* = 8 was further explored by constructing a N-J tree (Fig 4), coloured according to genepool designation at *K* = 8, as illustrated in previous figures. Good agreement was found between the N-J tree and InStruct results. The N-J tree clusters the majority of *spontaneum* accessions into a single clade, compromising of InStruct genepools *K*8_1 and *K*8_2. *K*8_4 (brown) forms a separate clade, with a substantial proportion of genepool *K*8_5 (dark blue); these *K*8_5 accessions are predominantly from India, Pakistan, and Nepal, with one accession from China, and none from Afghanistan or Iran. Genepools *K*8_6, *K*8_7 and *K*8_8 are largely grouped into the same clade. Accessions of *K*8_3 (yellow) are grouped together. *Agriocrithon* accessions were interspersed among the *vulgare* accessions.

The total genetic diversity (*H*_*E*_) of the InStruct genepools at *K* = 3 (S9 Table) show that *K*3_1 is most diverse, with a value of 0.712, and that *K*3_2 and *K*3_3 have lower diversity (0.549 and 0.507, respectively), which is consistent with *K*3_1 being mostly made up of *spontaneum*. The genetic diversity of each of the genepools under the *K* = 8 model (S10 Table), again shows that the genepools compromised mostly of *spontaneum* accessions are more diverse (*K*8_1—*H*_*E*_ = 0.638; *K*8_2—*H*_*E*_ = 0.729) than the genepools mostly containing *vulgare* and *agriocrithon* (*K*8_3 to *K*8_8; *H*_*E*_ 0.409 to 0.463).

Using Nei’s genetic distance measures, the relatedness between genepools at *K* = 8 was analysed (S11 Table). Comparisons between *K*8_1 and *K*8_2 (mostly *spontaneum*), gives a low value of 0.311, indicating greater similarity to each other. Comparisons between *K*8_1 and the six genepools predominantly made up of *vulgare* and *agriocrithon* (*K*8_3 to *K*8_8), give higher values (0.484 to 0.746), indicating more distant relationships. A similar range of values was obtained in comparisons between *K*8_2 and *K*8_3 to *K*8_8 (0.468 to 0.796). Comparing genepools *K*8_3 through to *K*8_8 to each other, *K*8_6, *K*8_7 and *K*8_8 are more closely related to each other (0.157 to 0.315) than to *K*8_3, *K*8_4 and *K*8_5 (0.409 to 0.878). *K*8_3 is most closely related to *K*8_5 (0.338), as is *K*8_4 (0.314).

### Geographical distribution of population structure in the three barley subspecies

Accessions belonging to the different InStruct genepools were geographically mapped according to their collection site coordinates. At *K* = 3 there is a clear distinction between *spontaneum* accessions located in the Near East and Central Asia (*K*3_1, pink; Figure B in S2 Fig) and *vulgare* accessions, *K*3_2 (green) and *K*3_3 (red) (Figure A in S2 Fig). The *vulgare* accessions are broadly divided into two genepools, with *K*3_2 being more prevalent in southern Eurasia (n = 169, 87% below 40°N) and *K*3_3 in more northerly latitudes (n = 150, 96% above 30°N). Both *vulgare* genepools are seen in Iran and Iraq but elsewhere they have divergent distributions. *K*3_2 predominates in Afghanistan, Pakistan, India, China and Japan, while *K*3_3 predominates in the Caucasus, Eastern Europe, Asiatic Russia and northeastern China. Some *spontaneum* accessions from Near East and Central Asia show admixture with *vulgare* genepools *K*3_2 and *K*3_3. The six *spontaneum* accessions from Tibet are assigned to *K*3_2 (Figure C in S2 Fig). Of the 23 *agriocrithon* accessions (Figure D in S2 Fig), only two are grouped in *K*3_1; the remainder fall within *K*3_2 (12) or *K*3_3 (9) and, along with the Tibetan *spontaneum*, their distribution reflects the geographic distribution of the *vulgare* found in the same region.

**Fig 5:**
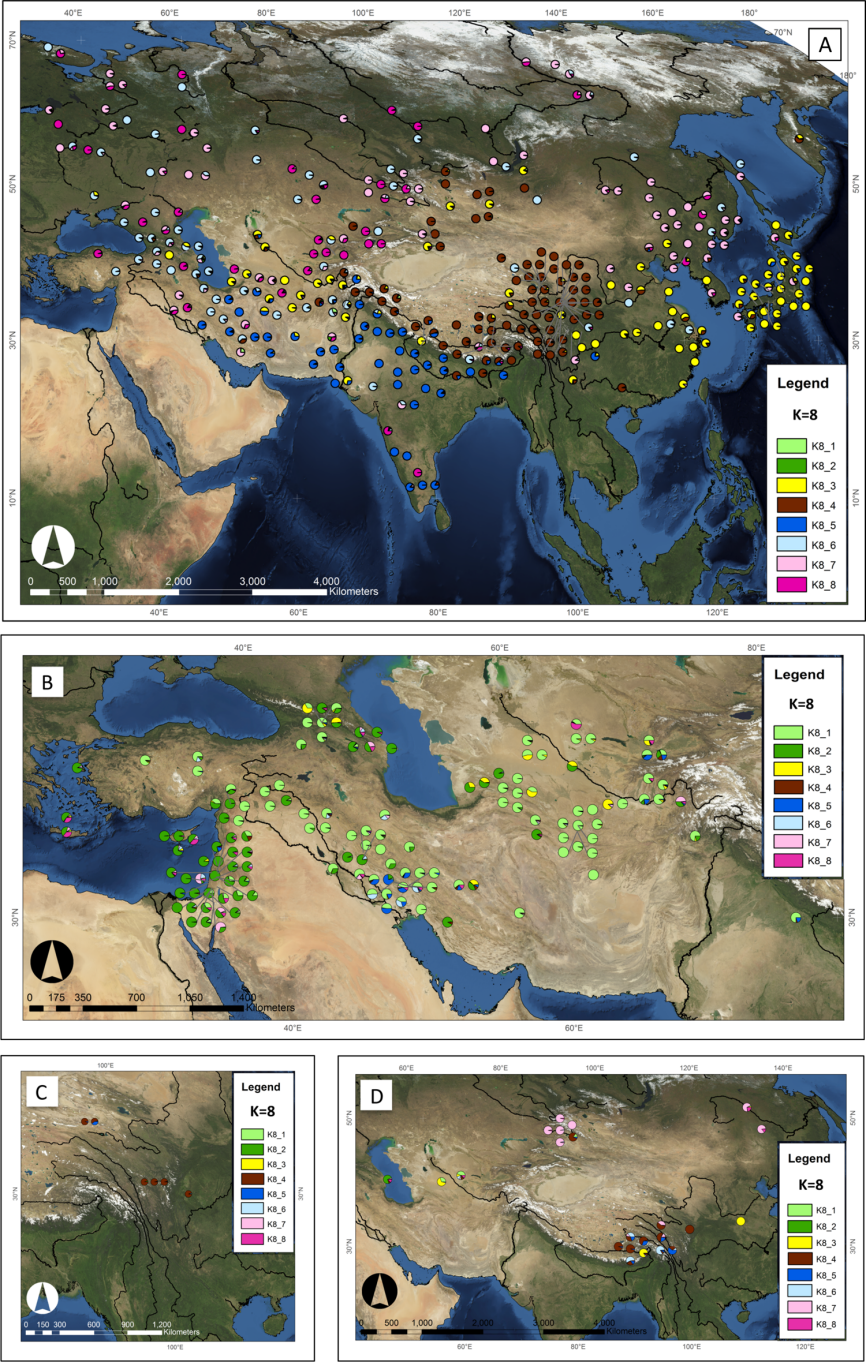
Geographical distribution of genepools of three barley taxa, according to the *K* = 8 model. Based on allele frequencies for 19 SSR markers in 351 *vulgare*, 142 *spontaneum* and 23 *agriocrithon* accessions. Each accession is depicted as a pie chart with the proportional membership of its alleles to each one of the eight genepools and was mapped according to its geographical coordinates. (A) *vulgare* (n = 351); *spontaneum* accessions (total n = 142) from (B) the Near East and Central Asia, and (C) Tibet; (D) *agriocrithon* accessions (n = 23). Maps generated using ArcMap v. 10.2, and NASA Blue Marble: Next Generation satellite imagery, which was produced by Reto Stöckli and obtained from NASA’s Earth Observatory (NASA Goddard Space Flight Center). See: http://earthobservatory.nasa.gov/Features/BlueMarble/.

The InStruct model at *K* = 8 provides additional geographic resolution (Fig 5), with *K*8_1 (light green) and *K*8_2 (dark green) largely composed of *spontaneum*, and *K*8_3 to *K*8_8 largely composed of *vulgare* and *agrocrithon* accessions.

We now consider each taxon in turn under the *K* = 8 model:

*Vulgare*: The *K*8_4 (brown) and *K*8_5 (dark blue) are more prevalent in southern Eurasia, and the *K*8_6 (pale blue), *K*8_7 (pale pink) and *K*8_8 (dark pink) genepools are broadly distributed across the Eurasian steppe (Fig 5A). Accessions from all six *vulgare* genepools identified at *K* = 8 are found together in the region between the westernmost end of the Tibetan Plateau and the Caspian Sea, including Afghanistan, Iran Uzbekistan, Tajikistan, and Kyrgystan. The accessions of *K*3_2 divide into three genepools: *K*8_3, *K*8_4 and *K*8_5. Genepool *K*8_3 (yellow, n = 69) is spread over a substantial part of southern Eurasia: it is the most common type in Japan (representing 90% of Japanese *vulgare*) and eastern China. Six accessions are also present around the edge of the Chinese Tibetan Plateau, along with one in Iran and five in Afghanistan. Members of this genepool are most closely related to those from *K*8_5 (dark blue) from pairwise diversity measures (S11 Table). All accessions carry the photoperiod responsive *Ppd-H1* allele, almost all (98%) are 6-rowed, 82% are hulled and 75% are winter-type. *K*8_4 (brown, n = 81) represents the predominant genepool of barley around the edge of the Tibetan Plateau. It consists of 94% naked caryopsis forms, is virtually all 6-rowed (97%), and predominantly carries the long-day photoperiod responsive *Ppd-H1* allele (89%) and winter SHG alleles (84%). From pairwise diversity measures (S11 Table), *K*8_4 is most closely related to *K*8_5. *K*8_5 (dark blue, n = 43) is most common in South Asia and Iran. All accessions were 6-rowed and long-day photoperiod responsive, 95% were hulled, and 82% spring SGH.

The *K*3_3 genepool also divides into three groups at *K* = 8. The geographic distribution of these three genepools, *K*8_6, *K*8_7 and *K*8_8, are similar to each other. From pairwise genetic diversity measures (S11 Table), these three genepools are more closely related to each other than to the rest of the *vulgare* accessions. *K*8_6 (pale blue, n = 59) is also spread throughout Eurasia. These barleys are almost all hulled (95%), 66% are 2-rowed, and have 59% spring types. Most (79%) have the non-responsive *ppd-H1* allele. *K*8_7 (pale pink; n = 65) is found throughout northern Eurasia, particularly in NE China, eastern Russia and in Central Asia. Barleys belonging to this group are 98% hulled, 79% are 6-rowed, and most are spring-type (88%) and photoperiod non-responsive (74%). Genepool Finally, *K*8_8 (dark pink, n = 34) occurs widely throughout Eurasia, from the south of India to the far north of Russia; members of this group are more common in northern Eurasia, but are rare in China. Nearly all members of this grouping are hulled (97%), 6-rowed (94%) and carry the photoperiod responsive *Ppd-H1* allele, while 74% are spring-type. Table 1 summarizes the above phenotype data for genepools at *K* = 8.

*Agriocrithon*: At *K* = 8 the 23 *agriocrithon* accessions show predominant membership to seven different genepools, showing relatedness to *vulgare* in their geographic vicinity (as also shown at *K* = 3; Fig 5D). Specifically, Tibetan Plateau *agriocrithon* predominantly belong to *K*8_4 (as do the *spontaneum* and the majority of the *vulgare* genepool in this region) and the others to genepools also found in *vulgare* in the same region (*K*8_3, *K*8_5 and *K*8_6). Five Xinjiang *agriocrithon* accessions belong to *K*8_7, and the remaining accession to *K*8_4. The two *agriocrithon* accessions from Heilongjiang, northeast China, belong to *K*8_7. The accession from Turkmenistan belongs to *K*8_3, from Azerbaijan belongs to *K*8_2, and from Uzbekistan to *K*8_1. These latter two are the only two *agriocrithon* accessions falling within *spontaneum* genepools (*K*8_1 and *K*8_2).

*Spontaneum*: At *K* = 8, most *spontaneum* accessions are split into two populations, *K*8_1 and *K*8_2, which partially overlap (Fig 5B). These genepools account for the majority of the *spontaneum* accessions analysed in this study and do not contain any *vulgare* accessions. *K*8_1 (pale green, n = 74) is predominantly found in Turkey, Iran, Iraq, Afghanistan and Central Asia; and *K*8_2 (dark green, n = 54) has a more westerly range in Israel, Jordan, Greece and Cyprus, and the west coast of the Caspian Sea. Many individuals across the range show admixture between the two *spontaneum* genepools, and between these genepools and *vulgare* genepools. In addition, a third *spontaneum* genepool (*K*8_4, n = 6) is found on the Tibetan Plateau (Fig 5C); these accessions belong to the same genepool as other *vulgare* and *agriocrithon* accessions in the region. A subset of *spontaneum* have ≥ 50% membership of *vulgare* genepools; this includes two accessions in the western Fertile Crescent that belong to *K*8_7, two accessions in southwest Iran that belong to *K*8_5, and several *spontaneum* accessions belonging to *K*8_3 in the Caucasus and Central Asia.

## Discussion

### The status of *agriocrithon* and Tibetan *spontaneum*

In the *K* = 8 model, the majority of *spontaneum* accessions split into two genepools (*K*8_1 and *K*8_2), which are distinct from the six *vulgare* genepools, showing a broad east-west division, with *K*8_1 predominating in the east and *K*8_2 in the west (Fig 5B); this mirrors the results reported by a number of authors using different genetic markers [31, 32, 62].

In the same model, all but two accessions of the 23 six-rowed brittle-rachised *agriocrithon* and all six *spontaneum* accessions from the Tibetan Plateau, do not fall within the two *spontaneum* genepools, but in the same genepools as the *vulgare* in their geographical vicinity (Fig 5), are more closely related to *vulgare* than non-Tibetan *spontaneum*. Thus, we infer that these accessions are mostly likely feral derivatives of local *vulgare* populations. *Agriocrithon* had previously been considered to be the progenitor of six-rowed cultivated barley (e.g. [2]). However, Tanno and Taketa [63] show that *agriocrithon* originates from hybridization between *spontaneum* and six-rowed *vulgare*, while Komatsuda et al. [64], proposed that *agriocrithon* originates from a back mutation in the brittle rachis genes (*btr1*, *btr2*) of 6-rowed *vulgare*. Clark [65] notes that *agriocrithon* has never been found in a truly natural habitat, but only in association with domesticated barleys.

There have been a number of recent genetic studies proposing that the Tibetan Plateau is one of the centers of domestication of cultivated barley [34, 35]. We, however, propose that the Tibetan *spontaneum* accessions analysed in this study, like *agriocrithon*, are also feral derivatives. A number of authors have questioned whether populations of wild barley in Tibet, Morocco and Ethiopia, i.e. outside of the Near East and Central Asia, are native, or introduced because of human activities or represent feral forms [33, 66, 67].

In determining the number and locations of domestication events using genetic analysis of domesticated plants and animals, and their wild progenitors, various authors (e.g. [68, 69]), have stressed how similar independent domestication and introgression can look. Introgression between domesticates and their wild progenitors and relatives can be a continuous process in regions where the two remain in close proximity to each other. Such processes can cause modern populations to appear as if they originated outside the regions where the initial domestication process occurred, and thus it can be erroneously concluded that multiple domestications have occurred [70]. This has been demonstrated in a study of maize and its wild progenitor teosinte, which found that introgression between the two impacted the inference of the region of domestication [71].

The genetic inference above concurs with the archaeobotanical data, which do not support an independent domestication of barley in Tibet, but instead show a clear west to east progression of radiocarbon dates associated with domesticated barley finds across Eurasia, as has been detailed above in the Introduction.

### Barley cultivation spread by several different routes across Eurasia

Our analysis of population structure in a set of extant 351 *vulgare* accessions suggests that prehistoric barley cultivation spread from the Near East by several different routes across Eurasia, possibly during different episodes in prehistory. Six different *vulgare* genepools show distinct phylogeographies (Fig 5) and consist of accessions with different morphological and phenotypic traits (Table 1).

**Fig 6:**
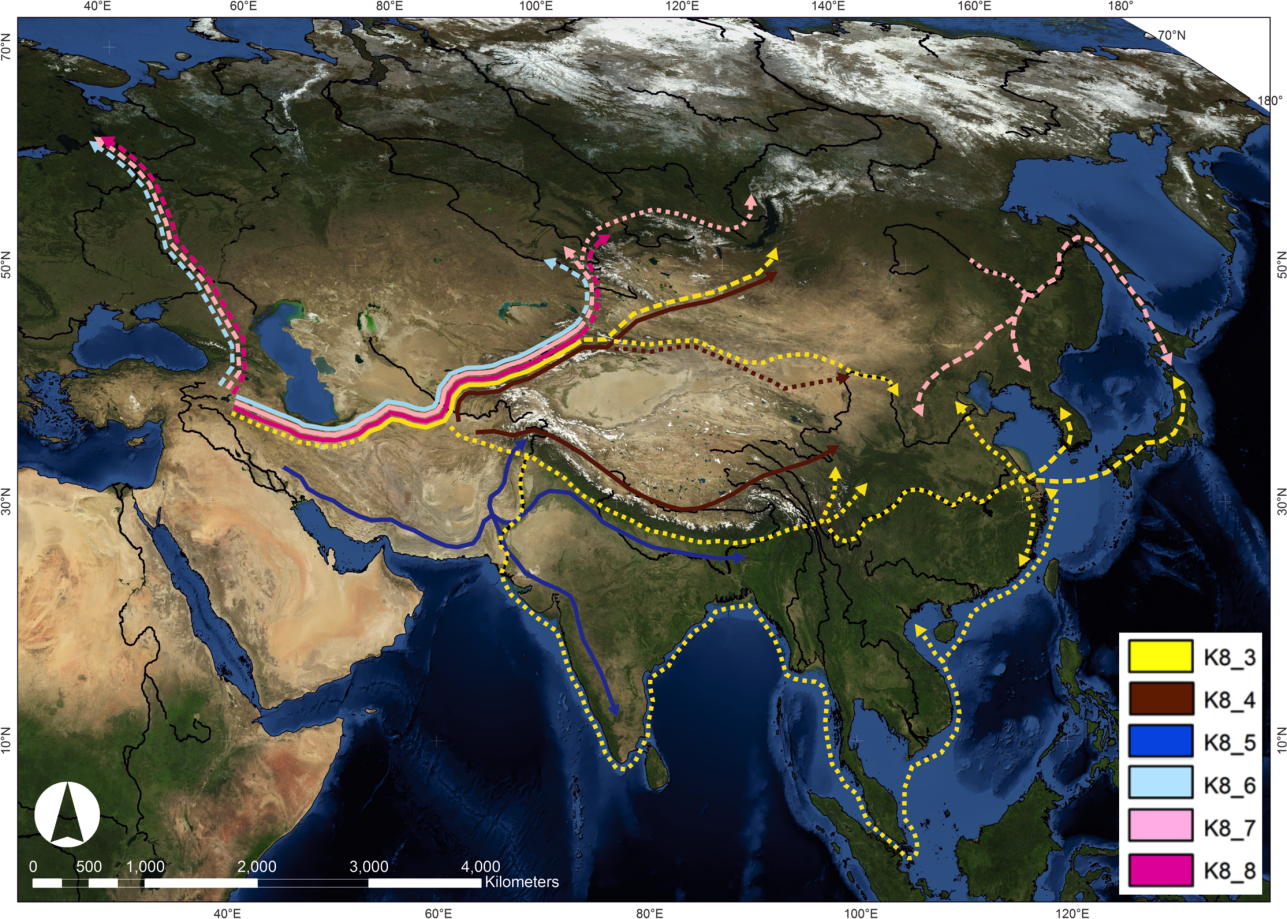
Proposed routes of spread of six *vulgare* genepools, according to the *K* = 8 model. Based on the geographical distribution of population structure in 351 *vulgare* accessions. Proposed routes of spread are indicated by solid or dashed lines, coloured according to the six *vulgare* genepools. Solid lines: barley genepool population structure clearly maps onto attested routes of agricultural spread. Dashed lines: routes of spread are more speculative, based on sparser distribution of barley genepools and/or archaeobotanical data. Map generated using ArcMap v. 10.2, and NASA Blue Marble: Next Generation satellite imagery, which was produced by Reto Stöckli and obtained from NASA’s Earth Observatory (NASA Goddard Space Flight Center). See: http://earthobservatory.nasa.gov/Features/BlueMarble/.

All six *vulgare* genepools are found together at the westernmost end of the Tibetan Plateau (Fig 5A). Within this region there are a number of possible routes into Central Asia through a series of mountain ranges, including the Hindu Kush, the Pamirs and the Karakorum. There is likely to have been another point of divergence further east in Afghanistan, where all but genepool *K*8_4 is found. Among these different genepools, there are three that are largely northerly in their distribution, two genepools more southerly and one that is more widespread. Fig 6 illustrates the potential routes of spread of each genepool, as will be discussed below in light of known archaeological and archaeobotanical evidence.

#### (1)–North and south of the Iranian plateau

The *K*8_5 (dark blue) genepool is the most common across the southern part of the Iranian Plateau, northern Afghanistan and throughout South Asia, and is limited to these regions. It appears to be distinct from the other five genepools, which are predominantly distributed along the northern Iranian Plateau, (*K*8_3, *K*8_4, *K*8_6, *K*8_7 and *K*8_8). This finding appears to confirm that the Iranian plateau was clearly a major geographical barrier to the dispersal of early farming societies. The likelihood that there were different routes is evident from the distinctive material cultures to the north and south of the plateau, as revealed by recent work at the sites of Sang-e Chakmaq and Djeitun in the north (c. 7^th^ to 5^th^ millennium BC) [72, 73], and Tappeh Rahmatabad, Tell–e Atashi and Mehrgarh to the south (8^th^ to 5^th^ millennium BC) [74].

#### (2) The Inner Asian Mountain Corridor (IAMC)

A number of authors, including Frachetti (who coined the phrase; [26]) and Spengler (e.g. [22]) have discussed the importance of the IAMC in the spread of domesticates east and west across Eurasia. Our results confirm this route’s importance, in that five out of the six *vulgare* genepools are distributed along the IAMC, the exception being the predominantly South Asian genepool *K*8_5 (Fig 6). The Near Eastern crop wheat is found in the IAMC around the mid of the 3^rd^ millennium cal. BC (e.g. Tasbas, 2617–2468 cal. BC; [27]) along with broomcorn millet [16], which has been shown to be domesticated in China and spread westwards towards South Asia and Europe during prehistory [75]. The oldest barley grains along the IAMC are from the middle of the 2^nd^ millennium cal. BC, in Ojakly in Turkmenistan (1,617–1,498 cal. BC), Tasbas in Kazakhstan (1,437–1,233 cal. BC) and Aigyrzhal-2 in Kyrgyzstan (1,630–1,497 cal. BC) [16, 76]. The distribution of these five genepools could reflect the expansion of barley cultivation through the IAMC in the 2^nd^ millennium BC.

#### (3) North of the Tibetan plateau—IAMC to the Tian Shan corridor

North of the Tibetan Plateau, the IAMC corridor leads eastwards into the Tianshan Corridor, in northwest China, part of a route that became the Chinese Silk Road in historical periods. In this study, very few *vulgare* landrace accessions were available from this region. However, archaeobotanical investigation and radiocarbon dates from Xinjiang attest to the dispersal of barley along this route during the 1^st^ millennium cal. BC, e.g. Sidaogou (975–831 cal. BC) and Yanghai (750–405 cal. BC) [15, 77]. These dates demonstrate that the spread of barley into China via Xinjiang happened substantially later than the spread of barley south of the Tibetan Plateau. Thus, these genepools distributed along the Tianshan Corridor may represent a later introduction of barley into China. Barley cultivation could also have travelled from the east during the 1^st^ millennium cal. BC, coming from the northeast Tibetan Plateau, where the oldest dated barley in China has been found in Qinghai (c. 2,000 cal. BC; [15, 28]). Wheat spread along this northern route during the early 2^nd^ millennium BC (e.g. Sidaogou, 1412–1127 cal. BC; Xicaozi, 1381–1047 cal. BC, both in Xinjiang [78]), around a thousand years earlier than barley [15, 27], attesting that the spread of wheat and barley into China via the IAMC and Tian Shan corridor was separated in both space and time.

We propose that two of the five *vulgare* genepools present in the IAMC *K*8_3 (yellow) and *K*8_4 (brown) could have been dispersed further eastwards along the north of the Tibetan Plateau in Xinjiang during the 1^st^ millennium cal. BC, though the distribution of landraces in this region is sparse. *K*8_3 is common in central China and Japan, while *K*8_4 remains limited to high altitude regions in the Altai and the Tibetan Plateau.

#### (4) South of the TP into China

During the period when the Indus civilization was established, the cultivation of both hulled and naked barley extended through northeastern Afghanistan, Pakistan, and in western India and as far east as Uttar Pradesh in the northwest. *K*8_5 (dark blue) is the most common genepool in South Asia. Its distribution closely mirrors that of the photoperiod responsive Haplotype D of the *PPD-H1* gene [37].

Genepool *K*8_5 is distributed along the southern boundary of the Himalayan uplift and towards southern India. Except for one individual in southern China, it has not spread further east than Assam in northeast India. Today barley is farmed in this region, however, and on into Myanmar and southwest China. The limited spread of *K*8_5 to the east is puzzling, and could reflect that this population is not adapted to high altitudes. It is also possible that there were cultural factors limiting its spread further east; for example, the Indus civilization was primarily a low altitude phenomenon, with relatively few settlements attested in uplands (C.A. Petrie, personal communication). We propose that this genepool reflects the dispersal of barley cultivation in South Asia during the mid 3^rd^ millennium cal. BC. Wheat travelled to a route south of the Tibetan Plateau, from Pakistan to the Indus and to the Ganges during the 3^rd^ millennium BC [27].

Genepool *K*8_4 (brown) is also distributed along the southern Tibetan Plateau, and shows a similar distribution to the photoperiod responsive Haplotype C of the *PPD-H1* gene [37]. This genepool is the most common across the Tibetan Plateau, and is also present in the Altai Mountains.

However, there is a clear geographical delineation between *K*8_4 and genepool *K*8_5 (dark blue), in that most *K*8_4 accessions are at high altitude (mean = 2,700 masl for those accessions where altitude data is available). Two other striking differences between these are that *K*8_4 accessions mostly have a naked caryopsis and a winter SGH, whereas most members of *K*8_5 are hulled and have a spring SGH. The naked caryopsis trait is strongly selected for because of its ease of processing [79], and barley of this type is the major staple in the Tibetan region. There is some evidence of admixture between *K*8_4 and *K*8_5 along the border of the plateau. The close relationship between *K*8_4 and *K*8_5 is mirrored in the N-J tree (Fig 4): *K*8_4 and those *K*8_5 accessions from India, Pakistan and Nepal, form a separate clade from all of the other genepools.

Liu et al. [15] discusses the possibility that naked barley was introduced onto the Tibetan Plateau initially from South Asia, via a route that remains to be identified. In our study, the distribution of genepool *K*8_4 suggests there are two possible scenarios for the distribution of this genepool onto the Tibetan Plateau: firstly at its western edge near Kashmir; secondly, at its southern edge near Bhutan. Evidence for the first scenario comes from the oldest date for barley in Kashmir (Kanispur, 2467–2236 cal. BC, [15]); however, there is no archaeology dated to the 3^rd^ and 2^nd^ millennium BC in this region and currently no archaeobotanical data available from the westernmost region of the Tibetan Plateau itself. Evidence for the second scenario is consistent with available radiocarbon dates for barley, with numerous direct dates in the 3^rd^ and 2^nd^ millennium cal. BC across the Ganges region (e.g. Damdama, 2832–2303 cal. BC; [80]); and barley has been dated on the southern Tibetan Plateau at the sites of Khog Gzung, c. 4,000 masl, and Bangtangbu at 3,700 masl (1393–1211 cal. BC and 1263–1056 cal. BC, respectively; [15]).

A conundrum in current archaeobotanical data is that the oldest directly dated barley grains in China are from the northeastern Tibetan Plateau, from four sites in eastern Qinghai province giving dates of c. 2,000 cal. BC, with the oldest from Xiasunjiazhai (2136–1959 cal. BC; [15, 28]). The fact that this date is significantly older than the existing direct dates from Xinjiang and also older than all available direct dates for barley in Central Asia, suggests that the source of the barley in Qinghai did not come via the IAMC [15]. Although the date for the Qinghai barleys is younger than the direct dates from Indian sites cited above, the lack of similar dates for barley along the southern Tibetan Plateau means that it is difficult to contextualise these early dates for barley in the northeastern Tibetan Plateau. Further archaeobotanical investigations would help clarify the precise routes and timings of barley’s spread onto the Tibetan Plateau and into East Asia.

The remaining genepool that may have spread along a trajectory south of the Tibetan Plateau is *K*8_3 (yellow), which shows a similar distribution to photoperiod responsive *PPD-H1* Haplotype G [37]. This genepool has a sparse distribution in the centre of our study region, but there are several accessions at the edge or on the Tibetan Plateau, which hint at a possible spread from the east of the Tibetan Plateau into central China, possibly following rivers eastwards to the coast.

#### 5) Northerly steppe route

In recent literature, there have been discussions about whether crops were dispersed across the vast Eurasian steppe, in an easterly direction for wheat and barley, and in a westerly direction for the Chinese millets.

In our study, there are three *vulgare* genepools with a largely northern distribution, *K*8_6 (pale blue), *K*8_7 (pale pink) and *K*8_8 (dark pink), scattered across northern Eurasia. These genepools are more closely related to each other than to the rest of the *vulgare* accessions, according to the PCAs (Fig 3) and the N-J tree (Fig 4), with considerable admixture between them. *K*8_6 and *K*8_7 are predominantly photoperiod non-responsive, while *K*8_8 is photoperiod responsive. The SGH is predominantly spring in all three genepools. The phylogeographic patterns suggest three possible routes:

(i) A dispersal northwards through the Caucasus towards the northern Eurasian steppe, followed by dispersals across northern Eurasia to the east and west. Barley has been found in Neolithic sites in the Caucasus, such as Göytepe, Azerbaijan [81], from the 6^th^ millennium cal. BC. (ii) A route eastwards along the IAMC and then north into the highland regions such as the Altai, with further dispersal into the steppe lands of the Russian Far East, with *K*8_7 predominating. (iii) A route eastwards across the vast Eurasian steppe at high latitudes, which has been called by others a steppe ‘highway’ (e.g. [82]).

Until recently, archaeobotanical evidence for farming in the Central Asian steppes and mountains has been sparse, and only documented after the Iron Age, ca. 800 BC (e.g. [22]). Frachetti and Spengler et al. [26, 83] have each argued against a trans-steppe highway during the Bronze Age, and instead propose a multi-regional emergence model, with cultures spreading to northerly latitudes from several regions in southern Central Asia. Naked caryopsis forms of barley predominate in Central Asia during this period [83]. This model fits with the results from Jones et al.’s study of the barley *PPD-H1* gene in European and Asian landraces [37], where there is a clear east-west divide between two photoperiod non-responsive haplotypes (A and B), with haplotype B making up the majority of non-responsive barleys in northerly NW Europe, and haplotype A making up the non-responsive haplotypes across temperate South and East Asia. This discontinuity in turn argues against a northerly steppe route as accounting for the initial spread of barley eastwards across Eurasia.

In this current study, however, more northerly *vulgare* accessions have been included as compared to Jones et al.’s study [37], which fill in some gaps in the north and centre of Eurasia. The population structure of these northerly genepools do not map neatly onto the *PPD-H1* haplotype data presented by Jones et al. [37]. In this current data, a discontinuity between western and eastern genepools across the Eurasian steppe is not visible; this could reflect intermingling of distinct economic traditions, which was thought to increase in the Iron Age [22], or over-stamping of earlier patterns by historic crop translocations, brought about by the Russians agriculturalists settling in Siberia during the 17^th^ century, or the construction of the Trans-Siberian railroad [84]. Barley in the three northerly genepools is over 95% hulled, which also suggests a later wave of barley cultivation over-stamping the Bronze Age distribution of predominantly naked caryopsis forms in Central Asia, as has been reported by [22].

#### (6) A maritime route

Another route by which barley could have travelled to East Asia is via what is called by some, for later periods, a maritime ‘Silk Route’ [85, 86]. Barley spread via a maritime route from the Indus civilization could explain the distribution of genepool *K*8_3 (yellow), which appears along the Indus River in South Asia, along the east coast of China, and in Korea and Japan. This is backed up by the broadly contemporaneous early dates for barley in Huangguasha, Fujian province, southeastern China (c. 2,000 BC; [87]); Korean Chulmun sites (c. 3,000–1,000 BC; [23]) and Japanese Jomon sites (>1,000 BC; [24]). During this period, agricultural practices in Korea and Japan were small-scale [23, 88]. The distribution of this genepool in Eurasia closely mirrors that of the photoperiod responsive *PPD-H1* Haplotype G [37]. Trade routes were also known to exist during later periods, such as the Han civilization, which had documented exchange with the Mauryan Empire in India, during the early part of the 1^st^ millennium century BC [85]. Thus, the patterns of distribution of *K*8_3 could reflect the spread of barley in different time periods.

The earliest directly dated barley in central and eastern China, however, comes from the 1^st^ millennium cal. BC, with the oldest date from Zhaogezhuang (895–791 cal. BC), Shandong Province, followed by Wangchenggang, Henan Province (764–516 cal. BC) [15]. As discussed above, genepool *K*8_3 could also have travelled to China via a route to the south of the Tibetan Plateau or north of the Tibetan Plateau, though the distribution of this genepool is sparse in both regions. These dates, along with textual evidence from oracle bones, for the cultivation of barley in central and eastern China, suggest that barley cultivation reached central/eastern China in the 1^st^ millennium cal. BC [15]. This would suggest barley in central and eastern China in this period may be derived from multiple sources, from both maritime and inland routes.

This predominately winter SGH and photoperiod responsive genepool *K*8_3 that is present in central and eastern China, and the majority of Japan, may have been selected for in agricultural regimes in East Asia, that double crop winter barley with a summer crop of rice [89]. Almost one fifth of the accessions in genepool *K*8_3 are naked barley and, of those, the majority originates in Japan, where naked barley forms an important cultural food.

#### (7) Two separate spreads into southern and northern Japan

There is evidence that two distinct barley genepools spread into Japan, one from the Russian Far East (predominantly *K*8_7; pale pink) and the other from eastern China (predominantly *K*8_3; yellow); individuals from *K*8_7 are in the northern-most Japanese island, Hokkaido, and the Russian territory of Sakhalin (Fig 3A), while most barley in Japan belongs to genepool *K*8_3. Leipe et al. [90] provide the earliest date yet for the cultivation of barley in Hokkaido, during the Okhotsk culture (ca. 440–890 cal. AD), considerably later than in southern Japan. This culture is thought to have spread to northern Japan through maritime contacts on Sakhalin and the Amur River region in the Russian Far East. Barley is believed to have been introduced into Kyushu, southern Japan, during the initial Yayoi period (ca. 1000–900 cal. BC; [91]). In a genetic study of broomcorn millet landraces using SSR markers, Hunt et al. [75] also shows evidence of two distinct routes of introduction of millet into the Japanese archipelago: the first in a southwesterly direction via the Korean peninsula and a second northeastern route into Hokkaido.

#### Diverse landscapes and environmental challenges

As barley spread through Eurasia, diverse landscapes, each with distinct ecological challenges, were encountered by the farmers and the crops they sought to cultivate. Two of the most challenging environments were the high Tibetan Plateau and the vast northern Eurasian steppe, which presented a complex range of challenges to plant physiology, e.g. short growing seasons, extremely low temperatures and low water availability.

On the Tibetan Plateau, genepool *K*8_4 is predominantly made up of naked caryopsis varieties that are photoperiod responsive and have a winter SGH varieties. This is an interesting result, as some have proposed that the acquisition of a spring SGH was necessary for barley to colonize the Tibetan Plateau (e.g. [22, 92, 93]). However, other authors confirm that winter types do grow at high altitudes on the plateau, at over 4,000 masl in Tibet [94, 95] and Nepal; at these elevations irrigation is often required [96]. Winter barley cultivation enables double cropping to take place, with either two crops of barley per year (one planted in October/November, the second in June/July), possibly with an additional summer crop such as buckwheat in between the two barley crops. Deep snow cover can insulate seedlings, enabling the plants to have a head start in the spring.

Spengler et al. [83] argues that photoperiod non-responsive and frost tolerant forms of barley would have been selected for at the lower latitude, elevated IAMC, thus pre-adapting varieties to the higher latitude, lower altitude regions of northern Central Asia, where photoperiod responsive types of barley would have been maladaptive. In this current study, there is a mixture of flowering time genotypes in the three northerly *vulgare* genepools: *K*8_6 and *K*8_7 are photoperiod non-responsive and *K*8_8 is photoperiod responsive, and the SGH is predominantly spring in all three. Knüpffer et al. [94] report a diversity of flowering time phenotypes in barley landraces growing in steppe regions, including some that are strongly photoperiod sensitive and having a medium to high vernalization requirements.

Our results, therefore, point to a complex pattern in extant *vulgare*, with a mixture of photoperiod responsive and non-responsive, and spring and winter SGH varieties, being successfully cultivated at northerly latitudes, as well as higher altitudes. It is this flexibility in growing seasons that allows barley cultivation to expand eastwards through different environments and different cropping regimes, as is documented, for example, in ancient Chinese texts [15]. In this study we have only considered two flowering time determinants: vernalization requirement and photoperiod response. There are other physiological attributes in crops grown at high altitudes and latitudes that need to be taken into account, including frost and cold tolerance, and early maturation [94]. Multi-cropping regimes influences the SGH, such as in East Asia, where barley with an extreme winter SGH is cultivated before a crop of rice in the spring [89].

#### Future prospects

This study has shown that archaeobotanical research, with direct radiocarbon dates for species of interest, along with phylogeographic patterns derived from extant landrace genetic analysis, can reveal routes of the spread of agriculture during prehistory. Further directly dated cereal remains, particularly from areas little studied, such as parts of Central Asia, will provide finer-scale details of the routes and timing of spread of crops across continents. In this study we have considered population structure using neutral microsatellite markers, and two sets of flowering time genes, which can inform us, to a limited degree, about environmental adaptation. In the age of genomics, SNP discovery will enable further investigations into population structure [97], while genomic scans are elucidating the genes involved in environmental adaptation [98]; understanding of the genomic basis of adaptation will not only help us understand the challenges facing early farmers as they spread across diverse landscapes, but will also help in predicting longer term climate change-mediated responses in crop plants, important for the food security of tomorrow [99].

## Conclusions

This study has shown that cultivated barley spread through Eurasia via several different routes, which were most likely separated in both time and space. The recently published direct radiocarbon dates provided by Liu et al. [15], along with previous published dates (e.g. [16, 28, 80]), have provided an invaluable framework with which to consider these phylogeographic patterns, as has an original paper by Zhao [25], who first proposed that a variety of routes were taken by farmers spreading eastwards into China.

We propose the following chronology for the spread of barley cultivation across Eurasia:

1. The IAMC
  Several barley genepools with different morphological features and flowering time genotypes spread through the IAMC in the 2^nd^ millennium cal. BC. From the IAMC barley dispersed further north and east in the 1^st^ millennium cal. BC.
2. A route to the south of the Tibetan Plateau
  A distinctive lowland genepool of barley spread eastwards to the south of the Iranian Plateau in the 5^th^ and 4^th^ millennium BC, and through South Asia, hugging the boundary of the plateau, with dates in northern Indian during the 3^rd^ millennium BC.
3. A maritime route from South Asia to China, Korea and Japan
  Although as yet unconfirmed, a maritime connection between the Indus civilization and coastal China could have brought barley into China from the 3^rd^ to 2^nd^ millennium cal. BC, with a possible later maritime route during the Han period, in the late 1^st^ millennium BC/early 1^st^ millennium AD. This genepool has a winter SGH, which may have been selected for to grow in rotation with a summer crop of rice.
4. A high altitude spread on the southern edge of the Tibetan Plateau
  A distinctive genepool, predominantly with a winter SGH and naked caryopsis, spread around the southern edge of the Tibetan Plateau, possibly entering the plateau from its western or southern end in the early 2^nd^ millennium cal. BC. This genepool is also in the northeastern plateau by c. 2,000 cal. BC.
5. A route along the northern edge of the Tibetan Plateau
  During the 1^st^ BC millennium cal. BC, two barley genepools were dispersed in Xinjiang to the north of the Tibetan Plateau, at least 1,000 years after the spread to the south of the Tibetan Plateau [15]. These genepools could have moved through the Tianshan Corridor from East to West, or from West to East.
6. A high latitude spread in the northern steppe
  Three predominately northern genepools, with different flowering time genotypes, dispersed northwards from southern Central Asia from the late 2^nd^ and early 1^st^ millennia cal. BC. A possible trans-steppe movement of barley occurred towards the end of this period, or during later historical periods.
7. A two-stage spread into Japan
  A northerly genepool spread into Hokkaido from the Russian Far East, in the mid to late 1^st^ millennium cal. AD. A different genepool spread into Japan from the south, during the late 1^st^ millennium cal. BC. These dates refer to substantive evidence of barley cultivation in Japan.

## Supporting information

S1 Fig Modelling of number of genepools in three barley taxa using InStruct. The results are based on allele frequencies for 19 SSR markers in 351 *vulgare*, 142 *spontaneum* and 23 *agriocrithon* accessions. *LnP(D)* and *∆K*, calculated according to [1], and implemented in CorrSieve 1.4 [2], are plotted against the number of modeled genepools (*K*). Dashed line = mean *LnP*(*D*), solid line = *∆K*. See S8 Table for values.
(TIF)

S2 Fig Geographical distribution of genepools of three barley taxa, according to the *K* = 3 model. The results are based on allele frequencies for 19 SSR markers in 351 *vulgare*, 142 *spontaneum* and 23 *agriocrithon* accessions. Each accession is depicted as a pie chart with the proportional membership each one of the eight genepools mapped according to its geographical coordinates. (A) *vulgare* (n = 351); *spontaneum* accessions (n = 142) from (B) the Near East and Central Asia, and (C) Tibet; (D) *agriocrithon* accessions (n = 23). Maps were generated using using ArcMap v. 10.2.
(TIF)

S3 Fig Phenotypes and genotypes of *vulgare* accessions analysed in this study. Each accession is depicted as a dot, and mapped using its geographical coordinates. For colour keys, see legends on each map. (A) Spike row-type (Hv_Row_2 = 2-rowed, Hv_Row_6 = 6-rowed; Hv_Row_ND = not determined). (B) Caryopsis type (Hv_Caryopsis_Hulled = hulled grains, Hv_Caryopsis_Naked = naked grains, Hv_Caryopsis_ND = not determined). (C) Predicted spring or winter SGH from PCR-based assays (Hv_SGH_S = spring growth habit, Hv_SGH_W = winter growth habit [4], Hv_SGH_ND = not determined). (D) Identify of the causative SNP in *PPD-H1*, as proposed by [3] (Hv_Ppd_C = wild type, flowering promoted in response to long days, Hv_Ppd_T = mutant type, flowering not promoted in response to long days, Hv_Ppd_ND = not determined). See S1 Text for assays for SGH and for *PPD-H1* genotyping. Maps generated using ArcMap v. 10.2, and NASA Blue Marble: Next Generation satellite imagery, which was produced by Reto Stöckli and obtained from NASA’s Earth Observatory (NASA Goddard Space Flight Center). See: http://earthobservatory.nasa.gov/Features/BlueMarble/.
(TIF)

S1 Table Details of germplasm accessions used in this study. Included in this study were 351 *vulgare*, 142 *spontaneum* and 23 *agriocrithon* accessions.
- Data concerning collection location, date of collection and taxon designation are as provided by the germplasm collections from which they were sourced.
- Germplasm collections: ICARDA: International Center for Agricultural Research in the Dry Areas, Beirut, Lebanon; IPK: Leibniz Institute of Plant Genetics and Crop Plant Research (IPK), Stadt Seeland, Germany; JIC: John Innes Centre, Norwich, UK; NordGen: Nordic Genetic Resource Centre, Alnarp, Sweden; NSGC: National Small Grains Research Facility, Idaho, USA; SCRI: Scottish Crop Research Institute (now the James Hutton Institute), Invergowrie, Scotland, UK; VIR: NI Vavilov Research Institute of Plant Industry, Saint-Petersburg, Russia.
- Genepool designation—*K* = 3, *K* = 8: Genepool with the highest proportional membership (>50%) ascribed in the *K* = 3 and *K* = 8 models using InStruct.
- Caryopis and row type–data was obtained from the germplasm collections from which they were sourced, and by visual inspection.
- *VRN-H1* multiplex assay for SGH - 1A, 5C = W; 1B, S = S. See S1 Text and [4].
- *ZCCT*—*VRN-H2* locus. 1 = presence of all 3 *ZCCT* genes (SGH = W), 0 = absence of 3 *ZCCT* genes (SGH = S). See S1 Text and [5].
- Determination of SGH—If a spring allele is observed in either the *VRN-H1* multiplex assay and/or *ZCCT* (*VRN-H2* locus) assay, the predicted SGH = spring (S). Both assays need to return a winter allele for SGH = winter (W) [4].
- *PPD-H1*- Identity of the causative SNP of the photoperiod response gene *PPD-H*, identified using Sanger sequencing or KASPar genotyping. C = wild type, flowering promoted in response to long days; T = mutant type, flowering not promoted in response to long days [3]. See S1 Text.
- Abbreviations: ND: not determined; SGH: seasonal growth habit; S: spring; W: winter.
(XLSX)

S2 Table Details of SSR markers used in this study. Sequence of primers, dye label. Primer sequences were kindly provided by J. Russell and L. Ramsay (James Hutton Institute).
(XLSX)

S3 Table Raw allele sizes for 19 SSR loci (expressed as relative sizes in bp) analysed in 351 *vulgare*, 142 *spontaneum* and 23 *agriocrithon* accessions. -9 = no data.
(XLSX)

S4 Table Q matrices–The individual Q-matrices generated for each accession using InStruct software, showing the membership coefficients to each population under the *K* = 3 and *K* = 8 models using InStruct. An individual was considered to be a member of the genepool with the highest probability (>50%). The results are based on allele frequencies for 19 SSR markers in 351 *vulgare*, 142 *spontaneum* and 23 *agriocrithon* accessions.
(XLSX)

S5 Table SSR marker diversity—The results are based on allele frequencies for 19 SSR markers in 351 *vulgare*, 142 *spontaneum* and 23 *agriocrithon* accessions, showing number of alleles per locus, the major *N* = number of accessions, *N*_*a*_ *=* number of alleles, = observed heterozygosity, *H*_*e*_ = expected heterozygosity, *F* = Fixation Index. (XLSX)

S6 Table Genetic diversities in *a priori* defined barley taxa: *vulgare*, *spontaneum* (including 6 Tibetan *spontaneum* accessions) and *agriocrithon*. SE = standard error, *N* = number of accessions, *N*_*a*_ ***=*** number of alleles, *H*_*o*_ = observed heterozygosity, *H*_*e*_ = expected heterozygosity, *F* = fixation Index. The results are based on allele frequencies for 19 SSR markers in 351 *vulgare*, 142 *spontaneum* and 23 *agriocrithon* accessions.
(XLSX)

S7 Table Pairwise diversity values between three barley taxa. *F*_*ST*_ (below diagonal) and *D* (above diagonal) values are based on the allele frequencies of 19 polymorphic SSRs. The results are based on allele frequencies for 19 SSR markers in 351 *vulgare*, 142 *spontaneum* and 23 *agriocrithon* accessions.
(XLSX)

S8 Table Modelling of number of genepools in three barley taxa using InStruct. The results are based on allele frequencies for 19 SSR markers in 351 *vulgare*, 142 *spontaneum* and 23 *agriocrithon* accessions. *LnP(D)* and *∆K*, calculated according to [1], and implemented in CorrSieve 1.4 [2]. Data is plotted in S1 Fig.
(XLSX)

S9 Table Genetic diversities in genepools at *K* = 3. The results are based on allele frequencies for 19 SSR markers in 351 *vulgare*, 142 *spontaneum* and 23 *agriocrithon* accessions. SE = standard error, *N* = number of accessions, *N*_*a*_ ***=*** number of alleles, *H*_*o*_ = observed heterozygosity, *H*_*e*_ = expected heterozygosity, *F* = fixation index.
(XLSX)

S10 Table Genetic diversities in genepools at *K* = 8. The results are based on allele frequencies for 19 SSR markers in 351 *vulgare*, 142 *spontaneum* and 23 *agriocrithon* accessions. *N* = number of accessions, *N*_*a*_ ***=*** number of alleles, *H*_*o*_ = observed heterozygosity, *H*_*e*_ = expected heterozygosity, *F* = fixation index.
(XLSX)

S11 Table Pairwise diversity values between genepools at *K* = 8. The results are based on allele frequencies for 19 SSR markers in 351 *vulgare*, 142 *spontaneum* and 23 *agriocrithon* accessions.
(XLSX)

S1 Text Supporting information text. (DOCX)

S1 File Raw genotyping data files generated using InStruct. Each *_f file contains a summary of the marginal posterior distribution of the parameters at a given value of *K*, per replicate.
(ZIP)
