# Supplementary figures and images for: Barley heads east: Genetic analyses reveal routes of spread through diverse Eurasian landscapes
Source: PLoS One. 2018 Jul 18;13(7):e0196652. doi: 10.1371/journal.pone.0196652 (PMC6051582; doi:10.1371/journal.pone.0196652)

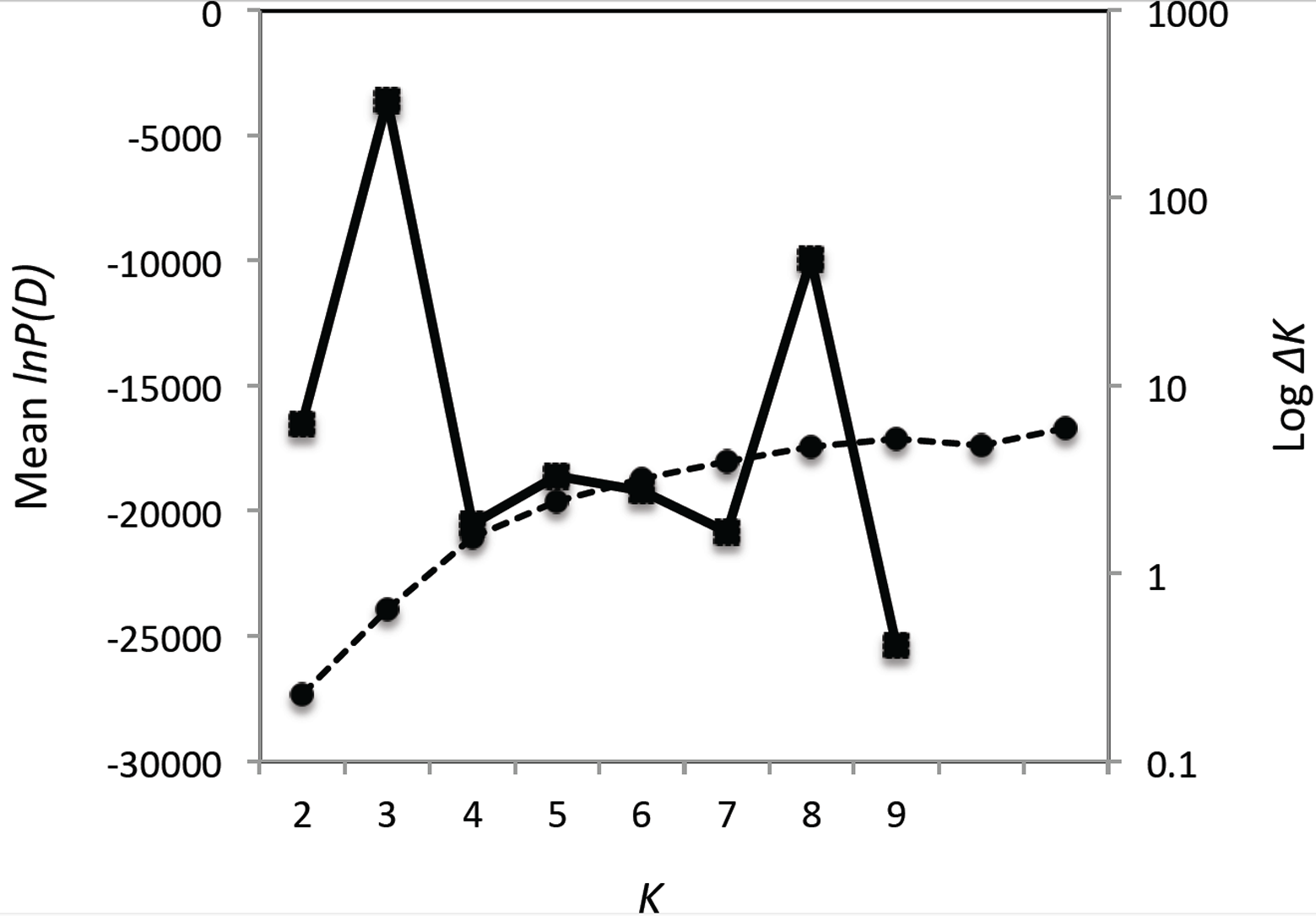

Supplement: S1 Fig — The results are based on allele frequencies for 19 SSR markers in 351 vulgare, 142 spontaneum and 23 agriocrithon accessions. LnP(D) and ∆K, calculated according to [1], and implemented in CorrSieve 1.4 [2], are plotted against the number of modeled genepools (K). Dashed line = mean LnP(D), solid line = ∆K. See S8 Table for values. (TIF) [file pone.0196652.s001.tif]

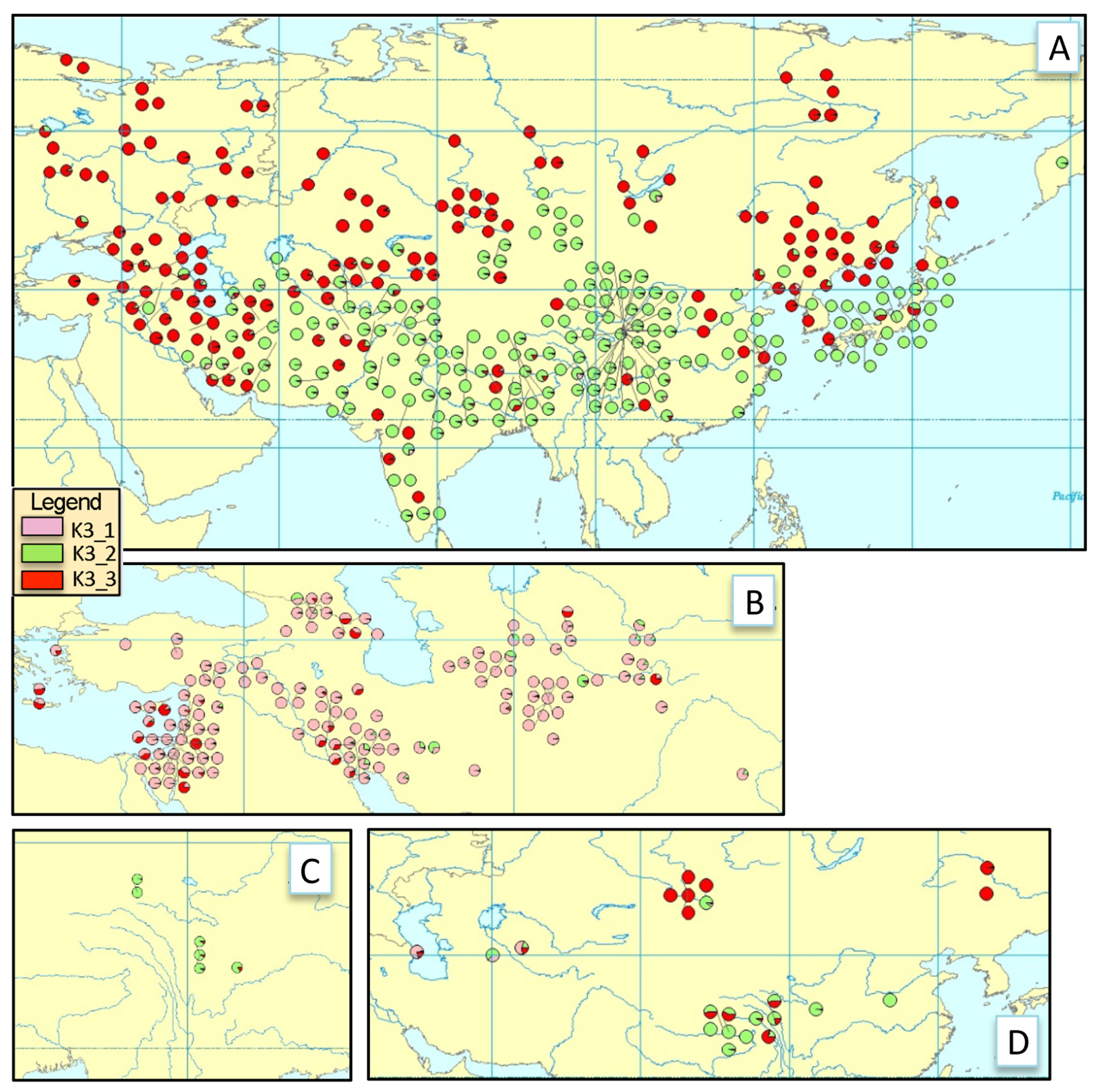

Supplement: S2 Fig — The results are based on allele frequencies for 19 SSR markers in 351 vulgare, 142 spontaneum and 23 agriocrithon accessions. Each accession is depicted as a pie chart with the proportional membership each one of the eight genepools mapped according to its geographical coordinates. (A) vulgare (n = 351); spontaneum accessions (n = 142) from (B) the Near East and Central Asia, and (C) Tibet; (D) agriocrithon accessions (n = 23). Maps were generated using using ArcMap v. 10.2. (TIF) [file pone.0196652.s002.tif]

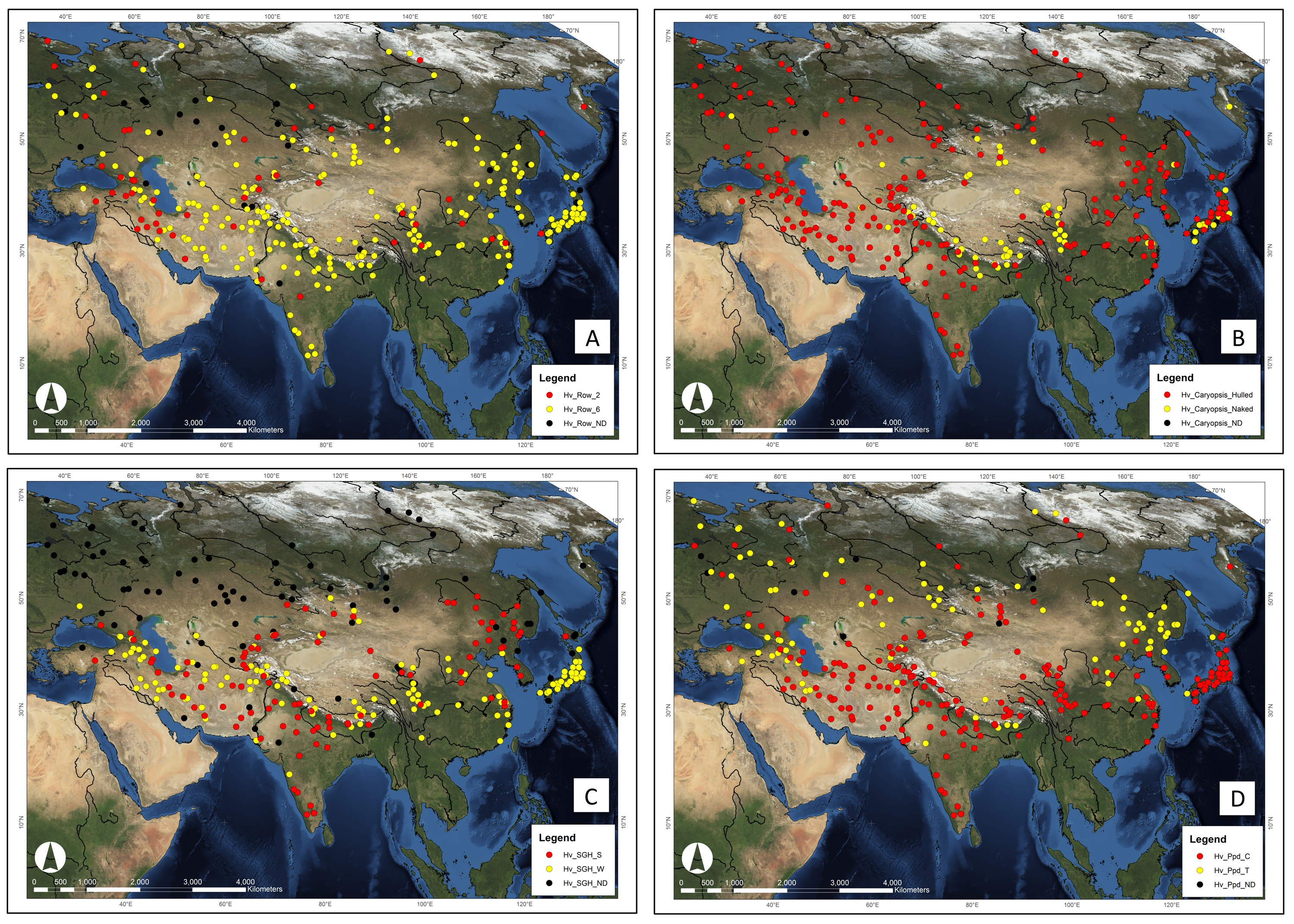

Supplement: S3 Fig — Each accession is depicted as a dot, and mapped using its geographical coordinates. For colour keys, see legends on each map. (A) Spike row-type (Hv_Row_2 = 2-rowed, Hv_Row_6 = 6-rowed; Hv_Row_ND = not determined). (B) Caryopsis type (Hv_Caryopsis_Hulled = hulled grains, Hv_Caryopsis_Naked = naked grains, Hv_Caryopsis_ND = not determined). (C) Predicted spring or winter SGH from PCR-based assays (Hv_SGH_S = spring growth habit, Hv_SGH_W = winter growth habit [4], Hv_SGH_ND = not determined). (D) Identify of the causative SNP in PPD-H1, as proposed by [3] (Hv_Ppd_C = wild type, flowering promoted in response to long days, Hv_Ppd_T = mutant type, flowering not promoted in response to long days, Hv_Ppd_ND = not determined). See S1 Text for assays for SGH and for PPD-H1 genotyping. Maps generated using ArcMap v. 10.2, and NASA Blue Marble: Next Generation satellite imagery, which was produced by Reto Stöckli and obtained from NASA’s Earth Observatory (NASA Goddard Space Flight Center). See: http://earthobservatory.nasa.gov/Features/BlueMarble/. (TIF) [file pone.0196652.s003.tif]
