# Supplementary material for: Barley heads east: Genetic analyses reveal routes of spread through diverse Eurasian landscapes
Source: PLoS One. 2018 Jul 18;13(7):e0196652. doi: 10.1371/journal.pone.0196652 (PMC6051582; doi:10.1371/journal.pone.0196652)
Supplement: S1 Text — (DOCX) [file pone.0196652.s015.docx]

**Barley heads east: genetic analyses reveal routes of spread through diverse Eurasian landscapes.**

**SUPPORTING INFORMATION TEXT**

**Methods**

SSR genotyping

PCR products were combined to form multiplexes of between one to three loci, and run on an ABI 3730 DNA Analyzer (Applied Biosystems). The resulting data were analysed using Genemapper 3.7 (Applied Biosystems), allowing relative SSR allele lengths to be recorded (starting at 2 for the smallest allele in markers with 2 bp repeats, and 3 with triplet repeats, etc). For SSR M12, two sets of alleles were recorded independently as SSR M12L (lower - smaller sized products) and SSR M12U (upper - which included alleles of greater length). With some markers heterozygosity was observed; this was most common in M13. Data was not included for accessions where ≥5 SSR markers failed. Missing alleles were scored as -9. All SSR data were treated as diploid in downstream analyses.

*PPD-H1* genotyping

The identity of the putative causative non-synonymous T+2036/C SNP (Thr → Ala) in *PPD-H1* exon 6 (SNP position relative to the start codon of GenBank accession AY943294. SNP also referred to as ‘SNP48’ [3, 6] was assayed using either:

(1) Sanger sequencing: A 1,369 bp genomic DNA fragment containing the target SNP in Exon 6 was PCR amplified. PCRs were performed in 10 µl reactions containing 1 x PCR buffer (Roche Diagnostics, Burgess Hill, UK), 1.5 mM MgCl_2_, 0.2 mM each dNTPs, 0.2 µM of both forward and reverse primers, 0.65 units Fast-Start *Taq* (Roche Diagnostics, Burgess Hill, UK), and 50 ng genomic DNA. PCR was performed using an Applied Biosystems 9700 PCR machine using the following cycling conditions: 94 ºC for 9 min, 35 cycles of 94 ºC for 45 s, 60 ºC for 45 s and 72 ºC for 1.5 min; and a final extension of 72 ºC for 10 min. Forward primer: GTGGCACTCAGGTATGCGAT, reverse primer: GTTGTTGCTGCTGCCGTTAG.

The resulting PCR products were Sanger sequenced in both directions using the PCR primers as sequencing primers.

(2) KASP SNP genotyping system (LGC Genomics, Hoddeson, U.K.): the KASP marker HvPPDH1_T2036C [4], was assayed using genomic DNA template (10 ng/µl) via a service contract at LGC Genomics. The resulting genotypic data was analysed using SNP Viewer v.1.99 (http://lgcgenomics.com/).

- In S1 Table

- C = cytosine, photoperiod responsive.

- T = thymine, photoperiod non-responsive.

Analysis of *VRN-H1* and *VRN-H2* alleles

(1) *VRN-H1* multiplex assay - details of the PCR-based assay are outlined in [4]. Amplicon banding profiles enable distinction between the *VRN-H1* haplotypes described in [7]. 1A, 5C = winter; 1B, S = spring.

(2) *VRN-H2* locus assay - The PCR–agarose gel assay determining the presence/absence of the three *VRN-H2* candidate genes (*ZCCT-Ha*, -*Hb*, and -*Hc*), as well as the closely linked *HvSNF2* gene (whose amplification acts as an internal positive control in the assay), has been previously described in [5]. Presence is confirmed by successful amplification of PCR products. 1 = presence of all 3 *ZCCT* genes (winter), 0 = absence of 3 *ZCCT* genes (spring).

Determination of SGH - If a spring allele is observed in either the *VRN-H1* multiplex assay and/or *ZCCT* (*VRN-H2* locus) assay, the predicted SGH = spring (S). Both assays need to return a winter allele for SGH = winter (W) [4].

**Results**

Hierarchical structure of Instruct groups

InStruct analysis revealed population structure among accessions with a degree of admixture between clusters. Analyses of *∆K*, *LnP(D)* and *Q*-matrix correlations indicate *K* values of *K*=3 and *K*=8 were significant (S8 Table and S1 Fig). Tracing the assignment of accessions to groups it can be seen that these two models relate to one another hierarchically. The majority of wild barley accessions are found in Group *K*3_2 (127; pink; 127) consisting of 125 *spontaneum* and two *agriocrithon* accessions. This group broadly subdivides into two groups *K*8_1 (pale green; 74) and *K*8_2 (dark green; 54) at *K*=8. Group K3_1 (green) comprises 215 accessions (including nine *spontaneum* and 12 *agrocrithon* accessions). These accessions broadly subdivide into three groups *K*8_3 (yellow; 76, including four *spontaneum* and three *agriocrithon* accessions), *K*8_4 (brown; 94, including six *spontaneum* and seven *agriocrithon* accessions) and *K*8_5 (dark blue; 46, including two *spontaneum* and one *agriocrithon* accession). Group *K*3_3 comprises 174 accessions, including eight *spontaneum* and nine *agriocrithon* accessions. These accessions broadly subdivide into three groups *K*8_6 (pale blue; 62, including three *agriocrithon* accessions), *K*8_7 (pale pink; 75, including three *spontaneum* and seven *agriocrithon* accessions) and *K*8_8 (dark pink; 35, including one *spontaneum* accession).

Phenotypic traits in *vulgare*

Phenotypic data for row-number (2- or 6-row) and caryopsis type (naked or hulled) were collated, where available, from passport data and visual inspection (S1 Table; S3 Fig). Two-rowed (50 accessions; 16% of those determined) is less common than six-rowed barley (269 accessions), and is scattered throughout Eurasia with a particular concentration in the western end of the study area (Figure A in S3 Fig). Naked barley (94 accessions; 27%) is less common than hulled (252 accessions) and predominates around the Tibetan Plateau, parts of Central Asia and Japan (Figure B in S3 Fig). Molecular determination of predicted SGH phenotype in *vulgare* accessions found 47% to be winter (148 accessions) and 53% to be spring (169 accessions), based on multi-locus haplotypes at the two major genetic loci controlling the trait (*VRN-H1* and *VRN-H2* – see Supplementary Information Methods for details of assay). Geographic mapping shows that barleys with a winter SGH are more prevalent in southern Eurasia below 45º latitude (representing 89% of all winter accessions), including mountainous regions in Iran, the edge of the Himalayas and the Tibetan Plateau (Figure C in S3 Fig). They are the predominant form in Japan, where winter forms represent 90% (27 of 30 accessions) of the *vulgare* investigated. Barleys with a predicted spring SGH are widely scattered, and are more prominent than winter types in the regions north of 30º (82% of accessions). Spring types also occur largely throughout the range of winter barleys, and are the predominant form in Eastern Russia. Molecular determination of long**-**day photoperiod response via genotype at the putative causative *PPD-H1* SNP T+2036/C found 70% (231 accessions) of *vulgare* to carry photoperiod responsive alleles (‘C’ nucleotide), while 30% (99 accessions) carried non- responsive alleles (‘T’). Long**-**day photoperiod responsive alleles are found throughout Eurasia, but predominate in the south, representing 89% (211 of 236 accessions) of all *vulgare* accessions below 45° latitude (including most of Iran, South Asia, Japan, and around the Tibetan Plateau) (Figure D in S3 Fig). Long-day non-responsiveness clearly predominates north of 30° latitude, where they represent 86% of *vulgare* accessions (96/112 accessions).

**References**

1. Evanno G, Regnaut S, Goudet J. Detecting the number of clusters of individuals using the software STRUCTURE: A simulation study. Mol Ecol. 2005;14. doi: 10.1111/j.1365-294X.2005.02553.x.

2. Campana MG, Hunt HV, Jones H, White J. CorrSieve: software for summarizing and evaluating Structure output. Molecular Ecology Resources. 2011;11(2):349-52. doi: 10.1111/j.1755-0998.2010.02917.x. PubMed PMID: WOS:000287200200014.

3. Jones H, Leigh FJ, Mackay I, Bower MA, Smith LMJ, Charles MP, et al. Population-based resequencing reveals that the flowering time adaptation of cultivated barley originated east of the fertile crescent. Molecular Biology and Evolution. 2008;25(10):2211-9. doi: 10.1093/molbev/msn167. PubMed PMID: WOS:000259327900013.

4. Cockram J, Norris C, O'Sullivan DM. PCR-Based Markers Diagnostic for Spring and Winter Seasonal Growth Habit in Barley. Crop Science. 2009;49(2):403-10. doi: 10.2135/cropsci2008.07.0398. PubMed PMID: WOS:000264654000004.

5. Karsai I, Szucs P, Meszaros K, Filichkina T, Hayes PM, Skinner JS, et al. The *Vrn-H2* locus is a major determinant of flowering time in a facultative X winter growth habit barley (*Hordeum vulgare* L.) mapping population. Theoretical and Applied Genetics. 2005;110(8):1458-66. doi: 10.1007/s00122-005-1979-7. PubMed PMID: WOS:000229718000013.

6. Lister DL, Thaw S, Bower MA, Jones H, Charles MP, Jones G, et al. Latitudinal variation in a photoperiod response gene in European barley: insight into the dynamics of agricultural spread from ‘historic’ specimens. Journal of Archaeological Science. 2009;36(4):1092-8. doi: 10.1016/j.jas.2008.12.012.

7. Cockram J, Chiapparino E, Taylor SA, Stamati K, Donini P, Laurie DA, et al. Haplotype analysis of vernalization loci in European barley germplasm reveals novel *VRN-H1* alleles and a predominant winter VRN-H1/VRN-H2 multi-locus haplotype. Theoretical and Applied Genetics. 2007;115(7):993-1001. doi: 10.1007/s00122-007-0626-x. PubMed PMID: WOS:000250071900010.
